# Enhanced Omega-3 Polyunsaturated Fatty Acid Contents in Muscle and Edible Organs of Australian Prime Lambs Grazing Lucerne and Cocksfoot Pastures

**DOI:** 10.3390/nu10121985

**Published:** 2018-12-15

**Authors:** Hung V. Le, Quang V. Nguyen, Don V. Nguyen, John R. Otto, Bunmi S. Malau-Aduli, Peter D. Nichols, Aduli E. O. Malau-Aduli

**Affiliations:** 1 Animal Genetics and Nutrition, Veterinary Sciences Discipline, College of Public Health, Medical and Veterinary Sciences, Division of Tropical Health and Medicine, James Cook University, Townsville, QLD 4811, Australia; vanhung.le@my.jcu.edu.au (H.V.L.); quang.nguyen2@my.jcu.edu.au (Q.V.N.); donviet.nguyen@my.jcu.edu.au (D.V.N.); john.otto@jcu.edu.au (J.R.O.); Peter.Nichols@csiro.au (P.D.N.); 2 National Institute of Animal Science, Thuy Phuong, Bac Tu Liem, Hanoi 129909, Vietnam; 3 College of Economics and Techniques, Thai Nguyen University, Thai Nguyen 252166, Vietnam; 4 Asia Pacific Nutrigenomics and Nutrigenetics Organisation (APNNO), CSIRO Food & Nutrition, Adelaide, SA 5000, Australia; bunmi.malauaduli@jcu.edu.au; 5 College of Medicine and Dentistry, Division of Tropical Health and Medicine, James Cook University, Townsville, QLD 4811, Australia; 6 CSIRO Oceans & Atmosphere, PO Box 1538, Hobart, TAS 7001, Australia; 7 Nutrition Society of Australia (NSA), Level 3, 33-35 Atchison Street, St Leonards, NSW 2065, Australia; 8 Australasian Section, American Oil Chemists Society (AAOCS), 2710 S. Boulder, Urbana, IL 61802-6996, USA

**Keywords:** lamb, *n*-3 LC-PUFA, muscle, liver, heart, kidney, rice bran, canola, cocksfoot, lucerne

## Abstract

The enhancement of health-beneficial omega-3 long–chain (≥C20) polyunsaturated fatty acid (*n*-3 LC-PUFA) contents in the muscle, liver, heart, and kidney of Australian prime lambs through pasture grazing and supplementation with oil infused pellets was investigated. Forty-eight first-cross prime lambs were randomly assigned into a split-plot design with pasture type as the main plot effect and pellet supplementation as a sub-plot effect in a feeding trial that lasted for nine weeks. The *n*-3 LC-PUFA content in *Longissimus dorsi* muscle of all lambs was well above the 30 mg threshold for “omega-3 source” nutrition claim under the Australian Food Standards and Guidelines. Pasture type impacted the fatty acid contents in muscle, heart, and kidney of prime lambs. Lambs grazing cocksfoot grass only had high 18:3n-3 (ALA) and *n*-3 LC-PUFA contents (67.1 mg/100 g and 55.2 mg/100 g, respectively) in the *Longissimus dorsi* muscle, which was not significantly different (*p* > 0.8990) from the contents of lambs grazing only lucerne. Supplementation of pellets with or without oil infusion to grazing lambs generally decreased the ALA and *n*-3 LC-PUFA contents and increased the *n*-6/*n*-3 ratio in the *Longissimus dorsi* muscle. The fatty acid content in the internal organs of grazing lambs was also affected by pellet supplementation. The liver and kidney of grazing lambs were both “good sources” (60 mg/100 g) of omega-3. The cocksfoot grass showed considerable potential for producing healthy, premium quality meat with high contents of *n*-3 and *n*-3 LC-PUFA, which may consequently enhance the omega-3 intake of Australian lamb consumers.

## 1. Introduction

Research on increasing the content of *n*-3 long-chain (≥C20) polyunsaturated fatty acids (*n*-3 LC-PUFA) in red meat has gained considerable attention because of their beneficial impact on human health. Omega-3 (*n*-3) fatty acids have potent anti-inflammatory and inflammation resolving properties in model systems [1] and *n*-3 fatty acid supplementation may be used as an effective tool in the primary and secondary prevention of cardiovascular disease [2,3]. In addition, many researchers have reported that *n*-3 fatty acids have therapeutic and protective effects on many types of cancers (breast, colorectal, leukaemia, gastric, pancreatic, oesophageal, prostate, lung, colon, head, and neck) [4,5,6,7]. 

Against the increasing recognition of health benefits derived from increased *n*-3 fatty acid consumption, recent studies have generally revealed that consumers do not obtain sufficient *n*-3 LC-PUFA for their daily recommended requirement. Sheppard and Cheatham [8] revealed that very few American children met even the lowest recommendations for eicosapentaenoic acid (EPA, 20:5n-3) and docosahexaenoic acid (DHA, 22:6n-3) intake. The findings of Pittaway et al. [9] showed that most healthy older adults in Tasmania, Australia, who participated in the observational study were unlikely to meet the recommended daily intake of 0.5 g EPA and DHA combined, without the use of fish oil supplements. In another study, Nichols et al. [10] found that future supplies of the beneficial *n*-3 LC-PUFA containing oils may be insufficient for the predicted increasing demands for their inclusion in livestock and aquaculture feeds, human foods, and nutraceutical products. Therefore, the utilization of alternative omega-3 LC-PUFA sources (such as red and other meat, egg, and milk) beyond marine products is of increasing importance in order to enhance *n*-3 LC-PUFA intake in humans. 

According to Howe et al. [11], lean red meat is an important natural food source of omega-3 LC-PUFA, the content of which can be manipulated by modifying the composition of livestock feeds. Australia was the world’s largest exporter of sheep meat and second largest producer of lamb and mutton in 2016–2017 [12]. Furthermore, Australians are among the highest lamb meat consumers in the world (9 kg of lamb per person in a year) [13]. Therefore, increasing omega-3 LC-PUFA content in lamb meat is one potential way to boost intake levels of omega-3 LC-PUFA among Australians, thereby meeting the daily-recommended requirements of these health-benefitting ingredients. In addition, lamb meat with high omega-3 LC-PUFA content helps to improve the reputation and competitiveness of Australian lamb meat in terms of healthy products.

Lamb production based on a grazing system has been reported to incur lower cost with respect to inputs, is more sustainable, and had greater amounts of health-claimable *n*-3 fatty acids such as EPA plus DHA than lamb production based on feedlot pellets, grain, or dry pasture/straw [14]. A number of current forage-types are presently used by the lamb industry; however, limited scientific information is available on how they influence *n*-3 LC-PUFA levels in lamb meat. There is a clear need to investigate the effect of these forage-types on *n*-3 LC-PUFA content in meat in order to assist lamb producers in selecting the optimal forage-type for producing premium lamb meat with health claimable sources of *n*-3 fatty acids. 

Cocksfoot cv. porto (*Dactylis glomerata* L. cv. Porto) was released in 1972 by the Tasmanian Department of Agriculture [15]. This grass grows actively in summer under low rainfall areas [16] and contains a high proportion of α-linolenic acid (ALA, 18:3n-3) [17,18], which is a precursor to the synthesis of the health claimable long-chain fatty acids in lambs [19,20]. Furthermore, cocksfoot cv. porto has a growth pattern which is better adapted to the Australian temperate climate and could replace ryegrass in relatively drier areas [15]. To the best of our knowledge, there is no published information on the fatty acid (FA) profile of cocksfoot cv. porto and its impact on the FA profile of lamb meat. Lucerne is a deep-rooted herbaceous perennial legume and is adapted to a broad range of agro-ecological environments [21]. Lucerne pasture has the potential to consistently produce premium grade carcasses and quality lamb meat with high levels of *n*-3 PUFA [20,22,23]. 

Supplementation of grains or a feedlot ration to grazing lambs in late spring and early summer is often necessary due to a decline in feed nutritive characteristics of annual pasture that occur as the plants mature [22] and also, in particular, under drought conditions. However, supplementation of grain or a feed concentrate to grazing lambs can affect the FA profile of lamb meat [24,25]. Canola oil was used for fattening lambs in an indoor system and successfully increased the *n*-3 LC-PUFA content in lamb meat [26]. Rice bran oil is rich in PUFA [27], and rice bran oil supplementation in an indoor system increased PUFA concentration in milk of dairy cows [28] and in adipose tissue of lambs [29]. Nevertheless, no published work is available on canola and rice bran oil supplementation in an external grazing system.

The internal organs including liver, heart, and kidney of lambs are nutrient dense animal-derived foods, which can provide protein, minerals (copper, iron, and zinc), and vitamins for humans [30,31]. Furthermore, these organs are also rich in the essential *n*-3 LC-PUFA content [32,33]. These edible organs can be directly consumed in some developing countries as cheap protein sources or processed to become traditional foods like liver pasties in developed countries such as France and Spain [34]. The study and potential enhancement of the nutritional composition of such organs could add value to animal by-products and earn extra income for the farming and slaughter sectors.

On the basis of the need in lamb production and emerging demand by consumers for healthy lamb meat as mentioned above, this study was designed to: (i) evaluate the potential of cocksfoot cv. porto grass to produce healthy, premium quality lamb with high content of health claimable *n*-3 LC-PUFA and (ii) to examine the effect of different pasture types and supplementation of lambs with pellets with or without plant oil infusion, on FA content in muscle and edible organs of Australian prime lambs.

## 2. Materials and Methods 

The study was conducted at the Tasmanian Institute of Agriculture’s Cressy Research and Demonstration Station, Burlington Road, Cressy, Tasmania, Australia from October to December, 2016. The use of animals and procedures performed in this study were all approved by the University of Tasmania Animal Ethics Committee (Permit No A0015657).

### 2.1. Animals, Diets, Experimental Design, and Feed Sample Collection

Experimental design: The experiment was a split-plot design with the basal pastures being cocksfoot cv. porto and lucerne. Main plot: Total of 1.5 ha of Cocksfoot cv. porto pasture with three equal 0.5 ha plots. Each 0.5 ha plot was split into four equal 0.125 ha sub-plots. Therefore, cocksfoot cv. porto pasture had 12 equal 0.125 ha sub-plots units in the main plot. Similarly, there were 12 equal 0.125 ha subplots as main plot units for lucerne pasture. These plots were used for rotational grazing. 

Sub-plots: Lambs were divided into four groups. Lambs in each group were allocated to one of the following four treatments: (1) cocksfoot cv. porto or lucerne pastures only as the control treatment; (2) cocksfoot cv. porto or lucerne pastures supplemented with: no oil pellets (NOP); (3) canola oil infused pellets (CO); and (4) rice bran oil infused pellets (RBO). Thus, each main plot unit had sub-plots.

Forty-eight White Suffolk x Corriedale first-cross prime lambs with an average liveweight (LWT) of 38.7 ± 0.7 kg weaned at six months were randomly allocated to 12 groups of four lambs balanced by gender. The 12 groups of lambs were allocated to six cocksfoot pasture subplots and six lucerne pasture subplots. The experimental lambs were grazed daily on pastures from 07:00 to 18:00 h and rotated to fresh pasture subplots every 14 days during the trial. Fresh water was available at all times throughout the grazing trial. The lambs in the supplemented treatments were individually offered oil infused pellets at 1 kg/head/day before going to pastures. The feeding trial lasted for nine weeks comprising three weeks of adjustment and six weeks of post-adjustment data collection.

Basal pasture and feed samples: the pasture samples were taken weekly from five area sites 50 cm × 50 cm of each subplot and then homogenized for withdrawing subsamples. The oil infused pellets were sampled from each bag and stored at −20 °C until the end of the trial.

### 2.2. Slaughter Protocol and Fatty Acid Analysis

All lambs were slaughtered at a commercial abattoir (Tasmanian Quality Meats, Cressy, Tasmania) adjacent to the experimental site after staying in the animal house for 12 h without feed and free access to fresh water. The slaughter procedures prescribed by the Meat Standards of Australia guidelines was strictly applied. Samples of liver, heart, and kidney were taken at the abattoir and immediately vacuum-sealed, code-labelled, and stored at −20 °C until FA analysis. Carcasses were chilled for 24 h at 4 °C before transporting to Robinson Meats, Glenorchy, Hobart, Tasmania, Australia. Thereafter, the *Longissimus dorsi* muscle were sampled at the 12/13 th rib of each carcass as a commercial loin chop (approximately 200 g) for subsequent FA analysis.

FA analysis was as described by Malau-Aduli et al. [33]. Briefly, the FA analysis included three processes. 1. A single-phase overnight extraction using CH_2_Cl_2_:MeOH:H_2_O (1:2:0.8 *v*/*v*) to extract total lipids from 1 gram of un-homogenised and wet liver, kidney, heart, and muscle tissues and feed samples according to a modified Bligh and Dyer protocol [35]. Phase separation with the addition of CH_2_Cl_2_:saline Milli-Q H_2_O (1:1 *v*/*v*) was carried out and followed by rotary evaporation of the lower CH_2_Cl_2_ phase at 40 °C to obtain the total lipids. 2. Methylation: An aliquot was taken from each total lipid extract for transmethylation with MeOH:CH_2_Cl_2_:HCl (10:1:1 *v*/*v*) for 2 h at 80 °C and Milli-Q H_2_O (1 mL) was then added before the FA methyl esters (FAME) extraction process with hexane:CH_2_Cl_2_ (4:1 *v*/*v*); extraction was performed three times. 3. Fatty acid quantification: Extracted FAME in glass vials were made up to a volume of 1500 μL with a known concentration of an internal injection standard (19:0). A 7890B gas chromatograph (GC) (Agilent Technologies, Palo Alto, CA, USA) equipped with an EquityTM-1 fused 15 m silica capillary column with 0.1 mm internal diameter and 0.1-μm film thickness (Supelco, Bellefonte, PA, USA), a flame ionisation detector, a split/splitless injector and an Agilent Technologies 7683 B Series autosampler was employed to analyse the FAME. The GC conditions were: splitless mode injection; carrier gas He; initial oven temperature 120 °C and then increased to 270 °C at 10 °C /min and to 310 °C at 5 °C /min. Peak quantification was performed using Agilent Technologies ChemStation software (Palo Alto, CA, USA). FA identifications were confirmed by GC-mass spectrometric (GC/MS) analysis with a Thermo Scientific 1310 GC coupled with a TSQ triple quadropole (Thermo Fisher Scientific, Milan, Italy) PTV injector and Thermo Scientific XcaliburTM software (Austin, Texas USA). The GC was equipped with a HP-5 cross-linked methyl silicone-fused silica capillary column (50 m × 0.32 mm internal diameter) which was of similar polarity to the column described above. The operating conditions was previously described by Miller, et al. [36] and helium served as the carrier gas. FA percentages (FA%) and contents (FA mg/100g) were calculated as follows [37], where 0.916 was the lipid conversion factor as cited by Clayton [38]. 

(a)  FA% = (individual fatty acid area) × (100)/(sum total area of fatty acids)(b)  FA mg/100 g = (Total lipid) × (LCF (0.916)) × ((%FA)/100) × 1000

### 2.3. Statistical Analysis

FA data were initially transformed into FA contents (mg/100 g). Thereafter, the data were analysed using the split-plot model in General Linear Model procedures (PROC GLM) of the Statistical Analysis System software (SAS Institute, North Carolina, USA) [39]. Pasture types were considered as the main plot effects and supplementation of pellets with or without oil infusion as subplot effects. Non-significant interactions between fixed effects were dropped from the analytical model and treatment differences were declared significant at *p* ≤ 0.05 using Bonferroni probabilities. Probability values ranging between *p* ≤ 0.056 and *p* ≤ 0.059 were deemed as “tending towards significance”.

## 3. Results 

The chemical composition of experimental diets is presented in Table 1. Dry matter (DM) of lucerne and cocksfoot were similar (*p* > 0.7880), while those of the pelleted supplements were much higher (*p* < 0.05) and ranged from 89.1% to 91.1%. Crude protein content of the different supplemented pellets ranged between 13.3% and 15.7%, which was lower (*p* < 0.05) than that in the basal lucerne feed (18.6%), but higher than in cocksfoot (13.3%). Acid detergent fibre (ADF) and Neutral detergent fibre (NDF) contents of the different supplemented pellets ranged from 6.8% to 8.0% and from 18.3% to 19.9%, respectively, while ADF and NDF content of the basal feed were 35.9% and 43.8%, respectively. In terms of EE content, the level in the supplemented pellets fluctuated between 4.6% and 4.9%, which was at least three-fold higher than the amount in the basal feed (1.8%). ME content of all supplemented pellets was approximately 12 MJ/kg, whilst the basal feed contained 9.5 (MJ/kg) ME.

### 3.1. FA Composition of Pastures and Supplementary Feeds

Table 2 shows the FA composition of supplementary feed and pastures. Cocksfoot cv. porto and lucerne pasture contained high proportions of ALA at 57.6 % and 51.9%, respectively. Supplementary feeds including NOP, CO and RBO had high levels of linoleic acid (LA, 18:2n-6) (50.3%, 32.9%, and 42.2%, respectively). A high relative level of 18:1n-9c was found in the NOP, CO and RBO treatments ranging from 24.5% to 44.5%. Cocksfoot cv. porto and lucerne pasture contained 73.1% and 69.7% of PUFA, respectively, while the PUFA proportion of the supplementary feeds varied from 39.2% to 54.5%. The *n*-3 PUFA levels of cocksfoot cv. porto and lucerne pastures were 58.0% and 52.2%, respectively, which were considerably higher than the *n*-3 PUFA levels of the three supplementary feeds. In contrast, NOP, CO, and RBO contained high relative levels of *n*-6 PUFA, ranging from 33.1% to 50.6%. The *n*-6/*n*-3 ratio of NOP and RBO diet treatments was similar and double the ratio of the CO treatment. The cocksfoot cv. porto and lucerne pastures had the lowest *n*-6/*n*-3 ratio (0.3) among all treatments.

### 3.2. Effect of Pellet Supplements on the Fatty Acid Contents in Longissimus dorsi Muscle, Liver, Heart, and Kidney

**Figure 1:**
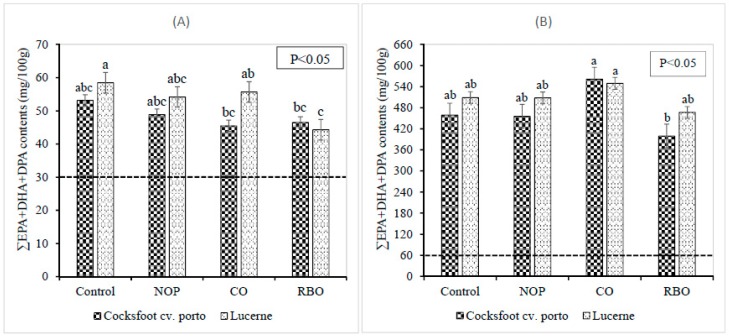
Effect of pellet supplementation on the contents of ∑EPA + DPA + DHA (EPA, eicosapentaenoic acid; DHA, docosahexaenoic acid; DPA, docosapentaenoic acid) in *Longissimus dorsi* muscle (**A**) and liver (**B**) of grazing lambs. Control: grazing on cocksfoot cv. porto or lucerne pastures only as basal pastures; NOP: basal pastures plus no oil pellets; CO: basal pastures plus canola oil infused pellets; RBO: basal pastures plus rice bran oil infused pellets. Different letters (a, b, c) indicate significant differences between treatments (*p* < 0.05).

FA of *Longissimus dorsi* muscle: Supplementation with pellets as depicted on Table 3, did not affect the total FA, MUFA, and PUFA contents in *Longissimus dorsi* muscle of grazing lambs. However, supplementation with pellets tended to decrease the ALA and *n*-3 PUFA contents in *Longissimus dorsi* muscle of grazing lamb, and the lowest values occurred in the RBO treatment. Lambs grazing on cocksfoot cv. porto or lucerne pastures only had similar ALA content (67.1 mg/100 g and 68.1 mg/100 g, respectively) in the *Longissimus dorsi* muscle (*p* > 0.899). Supplementation with NOP and RBO pellets increased the LA content in *Longissimus dorsi* muscle of lambs grazing on lucerne pasture. Lucerne grazing lambs supplemented with RBO had lower EPA and docosapentaenoic acid (DPA, 22:5n-3) contents in their *Longissimus dorsi* muscle than lambs grazing on lucerne pasture only. Pellet supplementation tended to decrease the total *n*-3 LC-PUFA and EPA + DHA + DPA contents (as demonstrated in Figure 1) in *Longissimus dorsi* muscle of lucerne grazing lambs and the lowest value occurred in the RBO treatment. Supplementation with pellets decreased the 18:0 content in the *Longissimus dorsi* muscle of lambs grazing cocksfoot cv. porto. The additional access to pellets by grazing lambs increased the *n*-6/*n*-3 ratio in the *Longissimus dorsi* muscle.

FA of liver: FA content of the liver are shown in Table 4. Pellet supplementation did not affect the SFA, MUFA, PUFA, *n*-3 PUFA, and *n*-3 LC-PUFA contents of grazing lambs. Supplementation of cocksfoot cv. porto with NOP and CO to grazing lambs tended to increase the ALA content in the liver. However, supplementation of pellets to lucerne grazing lambs did not change the ALA content in liver. Supplementation of CO to cocksfoot cv. porto grazing lambs resulted in higher EPA, DPA, PUFA, *n*-3 LC-PUFA, and EPA + DHA + DPA contents in liver in comparison with RBO supplementation. There was no difference in the EPA + DHA + DPA content of liver between grazing lambs with and without pellet supplementation (Figure 1).

FA of heart: FA contents of the heart are demonstrated in Table 5. Pellet supplementation did not change the FA content of lucerne grazing lambs. Nevertheless, supplementation of NOP and CO significantly decreased the ARA and DPA contents in the heart of cocksfoot cv. porto grazing lambs. Furthermore, NOP supplementation to cocksfoot cv. porto grazing lambs lowered the DHA and PUFA contents in heart tissues. There was no difference in the EPA + DHA + DPA content in heart of grazing lambs.

FA of kidney: Table 6 demonstrates the FA contents of the kidney. The FA contents in kidney of cocksfoot cv. porto grazing lambs were not affected by pellet supplementation. However, supplementation of NOP to lucerne grazing lambs significantly increased the *n*-6 PUFA, PUFA, and total FA of kidney tissues. Pellet supplementation did not change the EPA + DHA + DPA content in kidney of grazing lambs.

### 3.3. Effect of Pasture Types on the Fatty Acid Contents in Muscle, Liver, Heart, and Kidney

**Figure 2:**
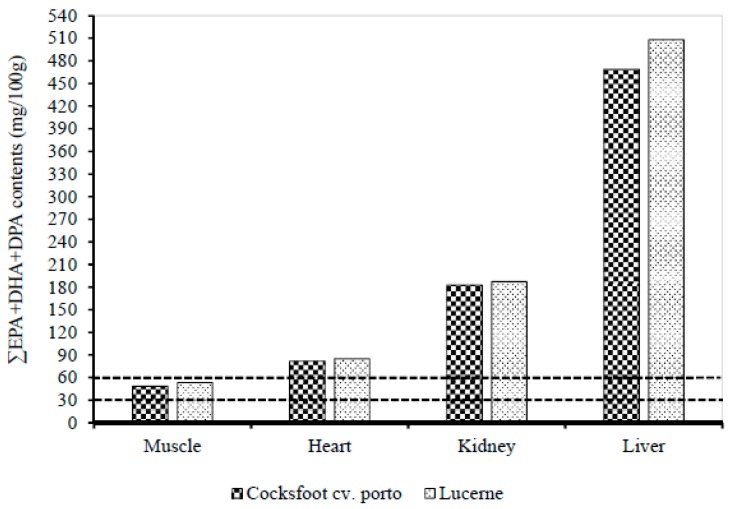
Effect of two different pasture types (cocksfoot cv. porto and lucerne) on the contents of ∑EPA + DPA + DHA in *Longissimus dorsi* muscle, heart, kidney, and liver. EPA, eicosapentaenoic acid; DHA, docosahexaenoic acid; DPA, docosapentaenoic acid. The lines at 30 and 60 mg/100 g represent “source” and “good source” of omega-3 FA respectively.

Table 7 shows the FA contents of the different internal organs of prime lambs as affected by the two different types of pastures. There was no significant difference in the FA content in liver of lambs grazing on different pasture types. The ALA, EPA, PUFA, *n*-3 PUFA, and *n*-6 PUFA contents in *Longissimus dorsi* muscle of lucerne grazing lambs were higher than that of cocksfoot cv. porto grazing lambs. There was no difference in the *n*-3 LC-PUFA and EPA + DHA + DPA contents in *Longissimus dorsi* muscle, liver, heart, and kidney of lambs grazing on cocksfoot cv. porto and lucerne pastures (Figure 2). The PUFA content in heart of lucerne grazing lambs (676.5 mg/100 g) was greater than that of cocksfoot cv. porto grazing lambs (640.6 mg/100 g). Lucerne grazing lambs had higher 20:3n-6 content in kidney than the cocksfoot cv. porto grazing lambs.

## 4. Discussion

### 4.1. FA of Pastures and Supplementary Feeds

The cocksfoot cv. porto and lucerne pastures in this study were abundant in ALA and total *n*-3 PUFA. Casey et al. [18] reported that cocksfoot pasture had 39.1% of ALA which is considerably lower than the result obtained in this study (57.6%). This could be attributed to the fact that the FA composition of pastures depend on many factors such as cultivar, cutting age, and season. Meľuchová, et al. [40] found that ALA concentration of pasture plants (mainly lucerne, grass, and herbs) decreased from 62% to 39% (of total FA) from May to August. Garcia et al. [41] also found that cultivar, cutting date, and season significantly influenced the FA composition, the ALA/LA ratio and PUFA. The relative level of ALA of lucerne pasture in this study was 51.9% which was similar to the finding of Wiking et al. [42] (53.5%) and doubled the ALA proportion in lucerne hay (22.1%) as reported by Nguyen et al. [26] in the same region. Glasser et al. [43] also found that the ALA proportion of fresh alfalfa was double that of alfalfa hay. The supplementary feeds used in the current study were rich in LA and total *n*-6 PUFA. Nguyen et al. [32] also found that 5% canola oil pellet contained high relative levels of LA and n-6 PUFA (26.7% and 27.4%, respectively).

### 4.2. Effect of Supplements on the Fatty Acid Contents in Longissimus dorsi Muscle, Liver, Heart and Kidney

*FA of Longissimus dorsi muscle:* Supplementation of omega-3 rich feed to lambs in indoor systems can increase the content of health benefit claimable FA in muscle [44]. However, unlike an indoor system, the response of FA content in muscle of grazing ruminants to supplements is not stable, and depends on the quality and quantity of pastures and supplements. Boughalmi and Araba [24] conducted a trial on the Timahdite lamb breed that revealed that lambs raised under pasture only had higher percentages of ALA and *n*-3 PUFA in the *semimembranosus* muscle than lambs did under the pasture and concentrate diet. Turner et al. [25] revealed that supplementation with whole cottonseed increased LA and the *n*-6/*n*-3 ratio and decreased ALA and *n*-3 PUFA in *Longissimus* muscle of Suffolk lambs and Katahdin lambs grazing on a grass–legume pasture. Ponnampalam et al. [45] found that adding oat grain at 245 g or at 175 g with flaxseed or 175 g with flaxmeal per day in the diet of grazing lambs increased the LA content and the *n*-6/*n*-3 ratio and did not affect *n*-3 PUFA and *n*-3 LC-PUFA content in the *Longissimus lumborum*, compared with lambs grazing pasture only. In addition, Fruet et al. [46] reported that beef cattle grazing on legume-grass pasture had higher concentrations of ALA in *Longissimus thoracis* muscle than those grazing on legume-grass pasture supplemented with whole corn grain at 1.4% of body weight. 

The results of the current study were in line with previous findings [24,25] that reported supplementation of pellets with or without oil infusion to grazing lambs led to a decrease in ALA and *n*-3 PUFA contents and increased the *n*-6/*n*-3 ratio in *Longissimus dorsi* muscle. The increase of the LA content in *Longissimus dorsi* muscle of lucerne grazing lambs supplemented with NOP and RBO pellets could be due to the high *n*-6 concentration of supplementary diets leading to more *n*-6 FA being digested, absorbed and finally incorporated in *Longissimus dorsi* muscle. The decrease of the 18:0 content in *Longissimus dorsi* muscle of cocksfoot cv. porto grazing lamb with pellet supplementation in this study was in agreement with the findings of Fruet et al. [46], that grass-fed beef had higher concentration of 18:0 when compared to grain-fed animals. The conversion of LA and ALA to their long-chain FA products share several of the elongation and desaturation enzymes and there was competition for incorporation into phospholipids between *n*-6 and *n*-3 FA [47]. Thus, the reduction of *n*-3 LC-PUFA and EPA + DHA + DPA contents in the *Longissimus dorsi* muscle of lucerne grazing lambs might be due to significant increases in LA content, and therefore, the competition for incorporation of *n*-6 and *n*-3 FAs into phospholipids. The lambs grazing cocksfoot cv. porto only had high contents of *n*-3 LC-PUFA (55.2 mg/100 g) in the *Longissimus dorsi* muscle, which was similar to that of only lucerne grazing lambs (60.4 mg/100 g) (*p* > 0.3150). The high content of *n*-3 LC-PUFA in lamb would be beneficial for meat consumers. According to Nichols et al. [10], the daily requirement per person was 500 mg of the LC omega-3 and a standard serve of red meat was 135 g under Australia and New Zealand regulation [48]. Therefore, consumers having two serves of cocksfoot cv. porto and lucerne grazing lamb meat (= 270 g) each day can meet about 30% of LC omega-3 daily requirement, which could result in a significant increase in LC omega-3 intake to Australians.

*FA of liver, heart and kidney*: The FA contents of organs (liver, heart, and kidney) can be affected by breeds and nutritional manipulation. Malau-Aduli et al. [33] reported that there were significant sire-breed variations in the FA content of kidney and muscle. Kashani et al. [49] found that Spirulina supplementation to lambs grazing on ryegrass pasture significantly increased the *n*-3 and *n*-6 PUFA composition in all organs (liver, heart, and kidney). The results of Nguyen et al. [32] demonstrated that there was no significant difference between liver FA profiles of 5% canola oil pellet-fed and control lambs in an indoor feeding system. This current study clearly demonstrated that supplementation with NOP and CO tended to increase the ALA content in the liver of cocksfoot cv. porto grazing lambs. The provision of NOP and CO supplements to grazing lambs resulted in adding more ALA to the lamb diet, which in turn, could explain the increased ALA content in the liver of cocksfoot cv. porto grazing lambs. In addition, among the supplemented treatments, the cocksfoot cv. porto grazing lambs with RBO supplementation had lower EPA, DPA, *n*-3 LC-PUFA, PUFA, and EPA + DHA + DPA contents of liver than those lambs in the CO treatment. This is likely due to the large difference in the ALA proportions of the CO (5.7%) and RBO treatments (2.7%). The competition for incorporation of *n*-6 and *n*-3 FAs into the phospholipids is a contributing factor, as previously discussed. The competition of incorporation of *n*-6 and *n*-3 FAs into the phospholipids also occurred in heart tissue, and could be the reason for the observed lowering of both ARA and DPA contents in heart of cocksfoot cv. porto grazing lambs with RBO and CO supplementation. The increase of the *n*-6 PUFA, PUFA, and total FA contents in kidney tissues of lucerne grazing lambs with NOP supplementation could also result from the high *n*-6 proportion (50.3%) of the NOP supplement. The kidney and liver of all lambs in this study contained high *n*-3 LC-PUFA contents (ranging from 163.2 mg/100 g to 572.6 mg/100 g), equal to and for many species over the *n*-3 LC-PUFA contents of wild Australian seafood such as fish, shellfish and lobster [10]. In addition, the *n*-6/*n*-3 ratio of liver and kidney (from 1.0 to 2.6) were well below the desirable ratio [50]. Therefore, the liver and kidney of grazing lambs could be considered as good sources of omega-3 [51].

### 4.3. Effect of Pasture Types on the Fatty Acid Contents in Muscle, Liver, Heart, and Kidney of Lambs

Pasture type did not affect the FA contents in liver of grazing lambs. However, pasture type impacted the FA contents in the muscle, heart, and kidney tissues. Lambs grazing on lucerne pasture had higher contents of ALA, 20:3n-6, DPA, PUFA, *n*-3 PUFA, and *n*-6 PUFA in *Longissimus dorsi* muscle compared with lambs grazing on the cocksfoot cv. porto pasture. In the present study, the fatty acid composition of cocksfoot cv. porto and lucerne pasture was similar, therefore, the difference in FA content in the *Longissimus dorsi* muscle of lambs grazing on these two types of pasture could be attributed to the distinctive characteristics of grass and legume pastures in terms of feed intake and the activity of stearoyl CoA desaturase enzyme. The findings of a meta-analysis conducted by Johansen et al. [52] revealed that cows grazing on legume species had 1.3 kg dry matter intake higher than cows grazing on grass species. Wiking et al. [42] found that transcription of stearoyl CoA desaturase in mammary tissue of cows grazing on high proportions of legume (white clover, red clover, and lucerne pasture) was significantly increased in comparison to cows fed maize/grass silage. Fraser et al. [23] also found that lambs finished on legume swards (red clover and lucerne) had significantly higher proportions of ALA in *Longissimus dorsi* muscle than lambs finished on perennial ryegrass sward. The *n*-3 LC-PUFA content in *Longissimus dorsi* muscle of lambs grazing on the lucerne and cocksfoot pastures (50.4 and 55.1 mg/100 g, respectively) were similar and well above the 30 mg cut-off point for “omega-3 source” claim under Australian guidelines [51]. This result could be due to the fact that *n*-3 LC-PUFA content in *Longissimus dorsi* muscle of grazing lambs was mainly synthesised from the ALA precursor [53]. It could also be due to the low elongation and desaturation of ALA into *n*-3 LC-PUFA, and the limited capacity of muscle lipids to incorporate *n*-3 LC-PUFA as occurs in ruminants [54]. Furthermore, cocksfoot cv. porto and lucerne pastures had similar proportions of ALA (57.6% vs. 51.9%, respectively). Ponnampalam et al. [45] performed a trial with lambs grazing on perennial lucerne and annual phalaris pasture, in which they also found no difference in the *n*-3 LC-PUFA content in muscle tissue of lambs grazing these two pasture types. 

## 5. Conclusions

Lambs grazing on lucerne pasture showed higher contents of ALA, 20:3n-6, EPA, PUFA, *n*-3 PUFA, and *n*-6 PUFA in *Longissimus dorsi* muscle in comparison with lambs grazing on the cocksfoot cv. porto pasture. All grazing lambs with or without supplements had high *n*-3 LC-PUFA content in *Longissimus dorsi* muscle (50.4 mg/100 g and 55.1 mg/100 g, respectively), which was well over the 30 mg cut-off point for labeling as a source of omega-3. A larger serve size, e.g., 135 or 150 g, as has been used in other studies, would see a good source of omega-3 (60 mg per serve) achieved. Lambs grazing on cocksfoot cv. porto pasture only also achieved high contents of ALA and *n*-3 LC-PUFA contents (67.1 mg/100 g and 55.2 mg/100 g, respectively), which was the same as those contents of only lucerne grazing lambs, with cocksfoot cv. porto clearly demonstrated to produce premium quality, healthy lamb meat, based on omega-3 PUFA content. Supplementation using pellets with or without oil infusion to grazing lambs generally decreased the ALA and *n*-3 PUFA contents and increased the *n*-6/*n*-3 ratio in *Longissimus dorsi* muscle. The addition of pellets to grazing lambs decreased the 18:0 content in *Longissimus dorsi* muscle of cocksfoot cv. porto grazing lambs. NOP and RBO supplementation increased the LA content in *Longissimus dorsi* muscle of lucerne grazing lambs. Pellet supplementation tended to reduce the EPA + DHA + DPA content in *Longissimus dorsi* muscle of lucerne grazing lambs. The fatty acid contents of internal organs of grazing lambs were affected by pellet supplementation. The *n*-3 LC-PUFA contents in the liver and kidney of grazing lambs were equal to the *n*-3 LC-PUFA contents of wild Australian seafood such as fish, shellfish, and lobster and can be considered and used as a good source of omega-3.

## Figures and Tables

**Table 1:** Proximate analysis of supplementary feed and pasture.

Chemical Composition (% DM)	Cocksfoot cv. Porto	Lucerne	NOP	CO	RBO
DM	20.5	20.7	89.1	91.1	90.2
CP	13.3	18.6	15.7	15.3	14.7
ADF	26.7	25.6	6.8	7.4	8.0
NDF	43.8	35.9	18.3	19.9	18.7
EE	3.0	1.8	2.1	4.6	4.9
Ash	6.4	6.8	4.0	6.5	5.0
%TDN	62.3	63.2	77.2	76.8	76.4
DE (Mcal/kg)	2.7	2.8	3.4	3.4	3.4
ME (MJ/kg)	9.4	9.5	11.7	11.6	11.5

Dry matter (DM), Neutral detergent fibre (NDF), Acid detergent fibre (ADF), Ether extract (EE) and crude protein (CP), Total digestible nutrients (%TDN), Metabolisable energy (ME). NOP was wheat-based pellet without infused oil. CO and RBO was wheat-based pellet infused with 50 mL/kg DM of oils from canola and rice bran sources, respectively. Total digestible nutrients (%TDN) were calculated as TDN (% of DM) = 82.38 − (0.7515 × ADF (% of DM)). Metabolisable energy (ME) was calculated by converting %TDN to digestible energy (DE (Mcal/kg) = %TDN × 0.01 × 4.4) which was converted as ME = (DE (Mcal/kg) × 0.82) × 4.185.

**Table 2:** Fatty acid composition (as % total fatty acids) of supplementary feeds and pastures.

% Lipid	Cocksfoot cv. Porto	Lucerne	NOP	CO	RBO
14:0	0.6	1.2	0.2	0.2	0.3
15:0	0.2	0.4	0.1	0.1	0.1
16:1n-9c	0.0	0.0	0.0	0.1	0.0
16:1n-7c	0.2	0.1	0.3	0.3	0.2
16:0	15.7	17.1	16.3	9.9	15.5
17:0	0.5	0.5	0.1	0.1	0.1
18:2n-6 LA	14.8	17.1	50.3	32.9	42.2
18:3n-3 ALA	57.6	51.9	3.5	5.7	2.7
18:1n-9c	1.0	0.5	24.5	44.5	32.3
18:1n-7c	0.2	0.1	1.0	2.6	1.2
18:1n-7t	0.0	0.0	0.0	0.0	0.0
18:0	0.1	2.2	0.3	0.5	0.3
20:4n-6 ARA	0.0	0.1	0.0	0.0	0.0
20:5n-3 EPA	0.0	0.0	0.1	0.1	0.1
20:3n-6	0.1	0.1	0.1	0.1	0.1
20:4n-3	0.1	0.1	0.2	0.1	0.1
20:2n-6	0.1	0.1	0.1	0.1	0.1
20:0	1.6	1.2	0.5	0.6	0.6
22:5n-6 DPA-6	0.0	0.0	0.1	0.1	0.1
22:6n-3 DHA	0.0	0.0	0.0	0.0	0.0
22:5n-3 DPA-3	0.0	0.0	0.1	0.0	0.0
22:0	1.0	1.3	0.6	0.3	0.4
23:0	0.3	0.5	0.1	0.0	0.1
24:0	0.9	1.4	0.3	0.2	0.3
∑SFA	20.9	25.8	18.5	11.8	17.6
∑MUFA	4.9	3.9	26.9	48.7	34.6
∑PUFA	73.1	69.7	54.5	39.2	45.5
∑*n*-3 LC-PUFA	0.1	0.1	0.3	0.3	0.3
∑*n*-3 PUFA	58.0	52.2	3.8	6.0	3.0
∑*n*-6 PUFA	15.2	17.5	50.6	33.1	42.5
∑other FA	1.0	0.4	0.1	0.2	2.3
*n*-6/*n*-3	0.3	0.3	13.3	5.5	14.2

LA, linoleic acid; ALA, α-linolenic acid; EPA, eicosapentaenoic acid; DHA, docosahexaenoic acid; DPA, docosapentaenoic acid; ΣSFA, total saturated fatty acids; ΣMUFA, total monounsaturated fatty acids; and total polyunsaturated fatty acids (ΣPUFA). ∑SFA is the sum of 14:0, 15:0, 16:0, 17:0, 18:0, 20:0, 21:0, 22:0, 23:0, 24:0; ∑MUFA is the sum of 14:1, 16:1n-13t, 16:1n-9, 16:1n-7, 16:1n-7t, 16:1n-5c, 17:1n-8+a17:0, 18:1n-9, 18:1n-7t, 18:1n-5, 18:1n-7, 18:1a, 18:1b, 18:1c, 19:1a, 19:1b, 20:1n-11, 20:1n-9, 20:1n-7, 20:1n-5, 22:1n-9, 22:1n-11, 24:1n-9; ∑PUFA is the sum of 18:4n-3, 18:3n-6, 18:2n-6, 18:3n-3, 20:3, 20:4n-3, 20:4n-6, 20:5n-3, 20:3n-6, 20:2n-6, 22:6n-3, 22:5n-3, 22:5n-6, 22:4n-6; ∑*n*-3 LC-PUFA is the sum of 20:5n-3, 20:4n-3, 22:6n-3, 22:5n-3; ∑*n*-3 PUFA is the sum of 18:3n-3, 18:4n-3, 20:4n-3, 20:5n-3, 22:6n-3, 22:5n-3; ∑*n*-6 PUFA is the sum of 18:2n-6, 18:3n-6, 20:4n-6, 20:3n-6, 20:2n-6, 22:5n-6, 22:4n-6; ∑other FA is the sum of other individual FA present at <0.1% except ARA, DHA, EPA, and DPA. All other abbreviations are as defined in Table 1

**Table 3:** Effect of pellet supplementation on IMF percentage (g fat/100 g) and fatty acid contents (mg/100 g) in *Longissimus dorsi* muscle tissue of grazing prime lambs * (LSM ± SE).

Items	Control		NOP		CO		RBO	
	CFP	Lucerne	CFP	Lucerne	CFP	Lucerne	CFP	Lucerne
IMF percentage	3.5 ± 0.5	3.4 ± 0.5	2.5 ± 0.5	2.5 ± 0.5	3.1 ± 0.5	2.7 ± 0.5	2.6 ± 0.5	3.6 ± 0.5
14:0	68.9 ± 13.9	54.5 ± 13.9	43.7 ± 13.9	65.7 ± 13.9	52.5 ± 13.9	39.5 ± 13.9	30.3 ± 13.9	49.6 ± 13.9
15:0	10.1 ± 1.7 ^a^	7.4 ± 1.7 ^ab^	5.5 ± 1.7 ^ab^	8.9 ± 1.7 ^a^	6.4 ± 1.7 ^ab^	5.9 ± 1.7 ^ab^	3.9 ± 1.7 ^b^	7.9 ± 1.7 ^ab^
16:1n-9c	9.0 ± 1.3 ^a^	6.2 ± 1.3 ^ab^	5.3 ± 1.3 ^ab^	8.4 ± 1.3 ^a^	5.6 ± 1.3 ^ab^	5.6 ± 1.3 ^ab^	4 ± 1.3 ^b^	7.3 ± 1.3 ^ab^
16:1n-7c	37.1 ± 7.7	31.8 ± 7.7	27.0 ± 7.7	41.9 ± 7.7	32.1 ± 7.7	30.7 ± 7.7	22.4 ± 7.7	35.0 ± 7.7
16:0	712.9 ± 113.8 ^ab^	541.3 ± 113.8 ^ab^	433.5 ± 113.8 ^ab^	753.2 ± 113.8 ^a^	545.9 ± 113.8 ^ab^	592.1 ± 113.8 ^ab^	416.6 ± 113.8 ^b^	663.9 ± 113.8 ^ab^
17:0	32.6 ± 5.5 ^ab^	24.9 ± 5.5 ^ab^	19 ± 5.5 ^bc^	36.3 ± 5.5 ^a^	21.2 ± 5.5 ^ab^	25.7 ± 5.5 ^ab^	16.6 ± 5.5 ^c^	32.9 ± 5.5 ^ab^
18:2n-6 LA	119.3 ± 8.7 ^bc^	122.8 ± 8.7 ^bc^	123.4 ± 8.7 ^bc^	156.8 ± 8.7 ^a^	110.6 ± 8.7 ^c^	144.2 ± 8.7 ^ab^	119.8 ± 8.7 ^bc^	157.2 ± 8.7 ^a^
18:3n-3 ALA	67.1 ± 5.5 ^ab^	68.1 ± 5.5 ^a^	33.9 ± 5.5 ^d^	57.3 ± 5.5 ^ab^	35.8 ± 5.5 ^cd^	51.6 ± 5.5 ^bc^	35.5 ± 5.5 ^cd^	39.5 ± 5.5 ^cd^
18:1n-9c	1169.0 ± 197.0 ^ab^	902.9 ± 197.0 ^ab^	767.5 ± 197.0 ^b^	1351.9 ± 197.0 ^a^	925.8 ± 197.0 ^ab^	1024.5 ± 197.0 ^ab^	759.6 ± 197.0 ^b^	1153.8 ± 197.0 ^ab^
18:1n-7c	41.2 ± 7.5 ^ab^	33.4 ± 7.5 ^b^	38.4 ± 7.5 ^ab^	54.1 ± 7.5 ^ab^	43.2 ± 7.5 ^ab^	50.3 ± 7.5 ^ab^	34.2 ± 7.5 ^ab^	55.5 ± 7.5 ^a^
18:1n-7t	83.6 ± 18.3 ^ab^	57 ± 18.3 ^b^	56 ± 18.3 ^b^	102.5 ± 18.3 ^ab^	67.5 ± 18.3 ^b^	82.9 ± 18.3 ^ab^	57.2 ± 18.3 ^b^	122.5 ± 18.3 ^a^
18:0	592.4 ± 75.4 ^a^	364.8 ± 75.4 ^bc^	328.4 ± 75.4 ^bc^	533.4 ± 75.4 ^ab^	362.5 ± 75.4 ^bc^	402.1 ± 75.4 ^abc^	307.3 ± 75.4 ^c^	515.2 ± 75.4 ^abc^
20:4n-6 ARA	33.7 ± 2.9	35.7 ± 2.9	38.2 ± 2.9	36 ± 2.9	34.8 ± 2.9	37.2 ± 2.9	34.5 ± 2.9	36.9 ± 2.9
20:5n-3 EPA	24.7 ± 1.9 ^abc^	26.9 ± 1.9 ^a^	22.1 ± 1.9 ^abc^	24.2 ± 1.9 ^abc^	19.8 ± 1.9^bc^	25 ± 1.9 ^ab^	20.2 ± 1.9 ^bc^	19.1 ± 1.9 ^c^
20:3n-6	6.1 ± 0.5 ^c^	6.7 ± 0.5 ^bc^	6.6 ± 0.5 ^bc^	8.0 ± 0.5 ^a^	5.8 ± 0.5 ^c^	7.7 ± 0.5 ^ab^	6.4 ± 0.5 ^bc^	7.5 ± 0.5 ^ab^
20:4n-3	2.0 ± 0.3	1.9 ± 0.3	2.2 ± 0.3	1.9 ± 0.3	1.7 ± 0.3	2.1 ± 0.3	1.9 ± 0.3	2.1 ± 0.3
20:2n-6	1.4 ± 0.2 ^bc^	1.1 ± 0.2 ^c^	1.5 ± 0.2 ^bc^	2.5 ± 0.2 ^a^	1.2 ± 0.2^c^	1.7 ± 0.2 ^bc^	1.9 ± 0.2 ^abc^	2.1 ± 0.2 ^ab^
20:0	4.4 ± 0.7	3 ± 0.7	3.3 ± 0.7	4.4 ± 0.7	3.9 ± 0.7	3.4 ± 0.7	2.7 ± 0.7	4.2 ± 0.7
22:5n-6 DPA-6	1.2 ± 0.2	1 ± 0.2	1.2 ± 0.2	1.4 ± 0.2	1.6 ± 0.2	1.2 ± 0.2	1.3 ± 0.2	1.2 ± 0.2
22:6n-3 DHA	6.7 ± 0.8	7.1 ± 0.8	5 ± 0.8	7.1 ± 0.8	7 ± 0.8	7.1 ± 0.8	6.1 ± 0.8	5.7 ± 0.8
22:5n-3 DPA-3	21.8 ± 1.1 ^abc^	24.5 ± 1.1 ^a^	21.7 ± 1.1 ^abc^	22.9 ± 1.1 ^ab^	18.8 ± 1.1^c^	23.7 ± 1.1 ^a^	20.2 ± 1.1 ^bc^	19.5 ± 1.1 ^c^
22:0	1.5 ± 0.1	1.6 ± 0.1	1.4 ± 0.1	1.5 ± 0.1	1.3 ± 0.1	1.4 ± 0.1	1.3 ± 0.1	1.5 ± 0.1
23:0	2.1 ± 0.1 ^a^	2 ± 0.1 ^a^	1.3 ± 0.1 ^c^	1.8 ± 0.1 ^ab^	1.4 ± 0.1 ^bc^	1.8 ± 0.1 ^ab^	1.5 ± 0.1 ^bc^	1.7 ± 0.1 ^abc^
24:0	2.3 ± 0.1 ^a^	2.2 ± 0.1 ^a^	1.8 ± 0.1 ^b^	2.1 ± 0.1 ^ab^	1.9 ± 0.1 ^ab^	2.1 ± 0.1 ^ab^	1.9 ± 0.1 ^ab^	2.1 ± 0.1 ^ab^
Total FA	3291.8 ± 467.8 ^ab^	2514.6 ± 467.8 ^ab^	2162.5 ± 467.8 ^ab^	3513.2 ± 467.8 ^a^	2496 ± 467.8 ^ab^	2762 ± 467.8 ^ab^	2070.8 ± 467.8 ^b^	3161.8 ± 467.8 ^ab^
∑SFA	1427.0 ± 208.0 ^a^	1001.5 ± 208.0 ^ab^	837.6 ± 208.0 ^ab^	1407.3 ± 208.0 ^a^	997 ± 208.0 ^ab^	1073.8 ± 208.0 ^ab^	782 ± 208.0 ^b^	1278.9 ± 208.0 ^ab^
∑MUFA	1469.1 ± 242.9 ^ab^	1138.6 ± 242.9 ^ab^	977.0 ± 242.9 ^ab^	1685.7 ± 242.9 ^a^	1162.8 ± 242.9 ^ab^	1297.6 ± 242.9 ^ab^	952.2 ± 242.9 ^b^	1485.8 ± 242.9 ^ab^
∑PUFA	294.7 ± 17.3 ^abcd^	306.2 ± 17.3 ^abc^	268.3 ± 17.3 ^bcd^	329.8 ± 17.3 ^a^	247.4 ± 17.3 ^d^	312.0 ± 17.3 ^ab^	257.4 ± 17.3 ^cd^	301.6 ± 17.3 ^abc^
∑*n*-3 LC-PUFA	55.2 ± 3.6 ^abc^	60.4 ± 3.6 ^a^	51.0 ± 3.6 ^abc^	56.0 ± 3.6 ^abc^	47.2 ± 3.6 ^c^	57.8 ± 3.6 ^ab^	48.3 ± 3.6 ^bc^	46.4 ± 3.6 ^c^
∑*n*-3 PUFA	123.2 ± 7.7 ^a^	129.8 ± 7.7 ^a^	85.5 ± 7.7 ^b^	114.4 ± 7.7 ^a^	83.1 ± 7.7 ^b^	110.2 ± 7.7 ^a^	84.1 ± 7.7 ^b^	86.1 ± 7.7 ^b^
∑*n*-6 PUFA	164.9 ± 10.8 ^bc^	170.9 ± 10.8 ^bc^	175 ± 10.8 ^bc^	208.8 ± 10.8 ^a^	157.4 ± 10.8 ^c^	196 ± 10.8 ^ab^	167.4 ± 10.8 ^bc^	209.2 ± 10.8 ^a^
∑other FA	99.6 ± 12.8	67.0 ± 12.8	78.3 ± 12.8	89.4 ± 12.8	87.6 ± 12.8	78.1 ± 12.8	78.8 ± 12.8	94.4 ± 12.8
*n*-6/*n*-3	1.4 ± 0.1 ^c^	1.3 ± 0.1 ^c^	2.1 ± 0.1 ^b^	1.9 ± 0.1 ^b^	1.9 ± 0.1 ^b^	1.8 ± 0.1 ^b^	2.0 ± 0.1 ^b^	2.4 ± 0.1 ^a^

* Values within the same row bearing different superscripts differ (*p* < 0.05); total FA is the combined FA contents; aside from Cocksfoot cv. Porto (CFP), least square mean (LSM), standard error (SE) and intramuscular fat (IMF), all other abbreviations are as defined in Table 1 and Table 2.

**Table 4:** Effect of pellet supplementation on total lipid percentage (g fat/100 g) and fatty acid contents (mg/100 g) in liver of grazing prime lambs (LSM ± SE) *.

Items	Control		NOP		CO		RBO	
	CFP	Lucerne	CFP	Lucerne	CFP	Lucerne	CFP	Lucerne
Lipid percentage	6.8 ± 0.4	6.9 ± 0.4	6.3 ± 0.4	7.0 ± 0.4	6.7 ± 0.4	6.7 ± 0.4	6.5 ± 0.4	6.5 ± 0.4
14:0	23.8 ± 5.0 ^ab^	21.4 ± 5.0 ^b^	38.4 ± 5.0 ^a^	22.7 ± 5.0 ^b^	28.4 ± 5.0 ^ab^	26.3 ± 5.0 ^ab^	22.6 ± 5.0 ^b^	17.7 ± 5.0 ^b^
15:0	12.9 ± 1.2 ^bc^	12.2 ± 1.2 ^c^	18.3 ± 1.2 ^a^	13.4 ± 1.2 ^bc^	15.9 ± 1.2 ^ab^	14.1 ± 1.2 ^bc^	13.4 ± 1.2 ^bc^	13.0 ± 1.2 ^bc^
16:1n-9c	22.2 ± 4.0	21.8 ± 4.0	31.1 ± 4.0	23.1 ± 4.0	24.8 ± 4.0	21.5 ± 4.0	21.2 ± 4.0	19.4 ± 4.0
16:1n-7c	33.9 ± 10.4 ^ab^	32.2 ± 10.4 ^ab^	60.2 ± 10.4 ^a^	29.6 ± 10.4 ^b^	36.5 ± 10.4 ^ab^	32.5 ± 10.4 ^ab^	28.0 ± 10.4 ^b^	26.4 ± 10.4 ^b^
16:0	723.7 ± 83.3	686.9 ± 83.3	879.1 ± 83.3	703.5 ± 83.3	786.6 ± 83.3	835.2 ± 83.3	660.6 ± 83.3	665.8 ± 83.3
17:0	51.3 ± 3.8 ^ab^	55.8 ± 3.8 ^ab^	57.4 ± 3.8 ^ab^	57.4 ± 3.8 ^ab^	56.5 ± 3.8 ^ab^	61.7 ± 3.8 ^a^	47.2 ± 3.8 ^b^	58.4 ± 3.8 ^ab^
18:2n-6 LA	370.2 ± 48.4	384.9 ± 48.4	394.2 ± 48.4	396.2 ± 48.4	419.6 ± 48.4	412.1 ± 48.4	327.0 ± 48.4	402.4 ± 48.4
18:3n-3 ALA	108.0 ± 22.8 ^b^	167.5 ± 22.8 ^ab^	180.0 ± 22.8 ^a^	162.1 ± 22.8 ^ab^	164.1 ± 22.8 ^ab^	184.8 ± 22.8 ^a^	106.2 ± 22.8 ^b^	109.0 ± 22.8 ^b^
18:1n-9c	928.1 ± 148.5	850.4 ± 148.5	1174.3 ± 148.5	800.9 ± 148.5	977.1 ± 148.5	950.8 ± 148.5	864.2 ± 148.5	807.2 ± 148.5
18:1n-7c	75.4 ± 15.0	67.7 ± 15.0	90.9 ± 15.0	66.3 ± 15.0	72.1 ± 15.0	73.0 ± 15.0	69.8 ± 15.0	71.8 ± 15.0
18:1n-7t	230.7 ± 43.5	237.5 ± 43.5	238.4 ± 43.5	213.8 ± 43.5	254.0 ± 43.5	240.4 ± 43.5	228.4 ± 43.5	217.2 ± 43.5
18:0	876.1 ± 65.8 ^ab^	859.9 ± 65.8 ^ab^	923.4 ± 65.8 ^ab^	808.2 ± 65.8 ^b^	918.5 ± 65.8 ^ab^	1023.0 ± 65.8 ^a^	786.0 ± 65.8 ^b^	869.3 ± 65.8 ^ab^
20:4n-6 ARA	260.6 ± 23.1	203.5 ± 23.1	211.9 ± 23.1	204.4 ± 23.1	240.2 ± 23.1	238.7 ± 23.1	200.1 ± 23.1	249.0 ± 23.1
20:5n-3 EPA	75.4 ± 14.4 ^bc^	117.2 ± 14.4 ^ab^	105.1 ± 14.4 ^abc^	108.8 ± 14.4 ^abc^	114.7 ± 14.4 ^ab^	117.7 ± 14.4 ^a^	68.1 ± 14.4 ^c^	82.2 ± 14.4 ^abc^
20:3n-6	31.6 ± 3.3 ^abc^	37.7 ± 3.3 ^abc^	30.3 ± 3.3 ^bc^	36.8 ± 3.3 ^abc^	39.4 ± 3.3 ^abc^	39.7 ± 3.3 ^ab^	29.7 ± 3.3 ^c^	40.4 ± 3.3 ^a^
20:4n-3	9.4 ± 1.3 ^b^	9.6 ± 1.3 ^b^	9.8 ± 1.3 ^b^	10.8 ± 1.3 ^ab^	11.4 ± 1.3 ^ab^	14.0 ± 1.3 ^a^	9.4 ± 1.3 ^b^	11.7 ± 1.3 ^ab^
20:2n-6	5.5 ± 0.8 ^b^	6.3 ± 0.8 ^ab^	5.0 ± 0.8 ^b^	5.8 ± 0.8 ^b^	8.1 ± 0.8 ^a^	7.1 ± 0.8 ^ab^	5.2 ± 0.8 ^b^	6.0 ± 0.8 ^ab^
20:0	6.0 ± 0.7 ^ab^	5.5 ± 0.7 ^ab^	6.0 ± 0.7 ^ab^	4.6 ± 0.7 ^b^	7.0 ± 0.7 ^a^	6.6 ± 0.7 ^ab^	5.4 ± 0.7 ^ab^	4.8 ± 0.7 ^b^
22:5n-6 DPA-6	9.2 ± 2.2	4.8 ± 2.2	9.3 ± 2.2	3.9 ± 2.2	5.1 ± 2.2	8.6 ± 2.2	9.7 ± 2.2	4.8 ± 2.2
22:6n-3 DHA	173.1 ± 26.2	132.8 ± 26.2	149.5 ± 26.2	156.8 ± 26.2	196.5 ± 26.2	207.8 ± 26.2	159.8 ± 26.2	165.9 ± 26.2
22:5n-3 DPA-3	210.5 ± 20.2 ^ab^	258.9 ± 20.2 ^a^	201.1 ± 20.2 ^ab^	242.5 ± 20.2 ^a^	249.9 ± 20.2 ^a^	223.8 ± 20.2 ^ab^	171.5 ± 20.2 ^b^	218.5 ± 20.2 ^ab^
22:0	8.5 ± 0.4 ^ab^	7.9 ± 0.4 ^b^	8.4 ± 0.4 ^b^	7.4 ± 0.4 ^b^	9.8 ± 0.4 ^a^	8.2 ± 0.4 ^b^	7.6 ± 0.4 ^b^	8.3 ± 0.4 ^b^
23:0	16.4 ± 2.2	18.3 ± 2.2	20.2 ± 2.2	21.3 ± 2.2	20.3 ± 2.2	22.1 ± 2.2	18.5 ± 2.2	22.2 ± 2.2
24:0	15.6 ± 0.9 ^ab^	15.1 ± 0.9 ^ab^	16.5 ± 0.9 ^ab^	14.4 ± 0.9 ^b^	17.7 ± 0.9 ^a^	16.2 ± 0.9 ^ab^	14.6 ± 0.9 ^b^	16.0 ± 0.9 ^ab^
Total FA	4662.0 ± 446.0	4580.7 ± 446.0	5334.2 ± 446.0	4476.4 ± 446.0	5075.9 ± 446.0	5183.5 ± 446.0	4246.8 ± 446.0	4480.4 ± 446.0
∑SFA	1734.2 ± 143.1 ^ab^	1682.7 ± 143.1 ^ab^	1967.7 ± 143.1 ^ab^	1652.8 ± 143.1 ^ab^	1860.5 ± 143.1 ^ab^	2013.2 ± 143.1 ^a^	1575.7 ± 143.1 ^b^	1675.3 ± 143.1 ^ab^
∑MUFA	1490.4 ± 231.0	1406.3 ± 231.0	1844.3 ± 231.0	1343.8 ± 231.0	1578.8 ± 231.0	1527.9 ± 231.0	1417.2 ± 231.0	1344.9 ± 231.0
∑PUFA	1318.3 ± 96.5 ^ab^	1376.1 ± 96.5 ^ab^	1369.8 ± 96.5 ^ab^	1376.9 ± 96.5 ^ab^	1509.0 ± 96.5 ^a^	1510.4 ± 96.5 ^a^	1137.4 ± 96.5 ^b^	1356.4 ± 96.5 ^ab^
∑*n*-3 LC-PUFA	468.4 ± 49.6 ^ab^	518.4 ± 49.6 ^ab^	465.4 ± 49.6 ^ab^	518.8 ± 49.6 ^ab^	572.6 ± 49.6 ^a^	563.3 ± 49.6 ^a^	408.7 ± 49.6 ^b^	478.3 ± 49.6 ^ab^
∑*n*-3 PUFA	582.6 ± 68.2 ^ab^	691.3 ± 68.2 ^ab^	658.0 ± 68.2 ^ab^	687.3 ± 68.2 ^ab^	744.4 ± 68.2 ^a^	753.2 ± 68.2 ^a^	520.5 ± 68.2 ^a^	593.5 ± 68.2 ^ab^
∑*n*-6 PUFA	708.9 ± 76.1	662.8 ± 76.1	685.7 ± 76.1	673.4 ± 76.1	741.1 ± 76.1	733.8 ± 76.1	597.5 ± 76.1	740.5 ± 76.1
∑other FA	118.5 ± 16.3	114.8 ± 16.3	150.2 ± 16.3	102.6 ± 16.3	126.7 ± 16.3	131.3 ± 16.3	115.0 ± 16.3	103.6 ± 16.3
*n*-6/*n*-3	1.2 ± 0.2	1.0 ± 0.2	1.1 ± 0.2	1.0 ± 0.2	1.0 ± 0.2	1.1 ± 0.2	1.2 ± 0.2	1.3 ± 0.2

* Values within the same row bearing different superscripts differ (*p* < 0.05); all other abbreviations are as defined in Table 1, Table 2 and Table 3.

**Table 5:** Effect of pellet supplementation on total lipid percentage (g fat/100 g) and fatty acid contents (mg/100 g) in heart of grazing prime lambs (LSM ± SE) *.

Items	Control		NOP		CO		RBO	
	CFP	Lucerne	CFP	Lucerne	CFP	Lucerne	CFP	Lucerne
Lipid percentage	2.3 ± 0.1	2.3 ± 0.1	2.4 ± 0.1	2.3 ± 0.1	2.4 ± 0.1	2.3 ± 0.1	2.3 ± 0.1	2.4 ± 0.1
14:0	6.0 ± 2.1	5.7 ± 2.1	9.5 ± 2.1	5.7 ± 2.1	3.9 ± 2.1	6.7 ± 2.1	7.7 ± 2.1	5.9 ± 2.1
15:0	2.6 ± 0.5	2.6 ± 0.5	3.2 ± 0.5	3.0 ± 0.5	2.5 ± 0.5	3.1 ± 0.5	3.3 ± 0.5	2.7 ± 0.5
16:1n-9c	2.7 ± 0.4	2.8 ± 0.4	3.1 ± 0.4	2.7 ± 0.4	2.4 ± 0.4	2.6 ± 0.4	3.4 ± 0.4	2.4 ± 0.4
16:1n-7c	5.2 ± 0.9	5.1 ± 0.9	6.4 ± 0.9	5.0 ± 0.9	4.2 ± 0.9	4.8 ± 0.9	6.7 ± 0.9	4.9 ± 0.9
16:0	184.4 ± 13.4	197.6 ± 13.4	193.9 ± 13.4	196.5 ± 13.4	168.5 ± 13.4	187.7 ± 13.4	202.1 ± 13.4	194.9 ± 13.4
17:0	13.3 ± 1.6	15.6 ± 1.6	14.6 ± 1.6	15.4 ± 1.6	12.2 ± 1.6	15.3 ± 1.6	15.9 ± 1.6	14.0 ± 1.6
18:2n-6 LA	390.0 ± 24.4	416.4 ± 24.4	357.8 ± 24.4	415.7 ± 24.4	392.6 ± 24.4	401.2 ± 24.4	371.0 ± 24.4	400.8 ± 24.4
18:3n-3 ALA	33.0 ± 10.2	55.2 ± 10.2	53.9 ± 10.2	53.8 ± 10.2	33.5 ± 10.2	35.0 ± 10.2	54.4 ± 10.2	52.6 ± 10.2
18:1n-9c	208.2 ± 34.6	205.4 ± 34.6	239.7 ± 34.6	192.0 ± 34.6	200.8 ± 34.6	195.4 ± 34.6	257.4 ± 34.6	187.3 ± 34.6
18:1n-7c	40.6 ± 3.4	37.3 ± 3.4	31.8 ± 3.4	38.8 ± 3.4	36.8 ± 3.4	36.4 ± 3.4	38.0 ± 3.4	33.0 ± 3.4
18:1n-7t	44.3 ± 6.3	46.2 ± 6.3	41.8 ± 6.3	46.7 ± 6.3	51.8 ± 6.3	49.4 ± 6.3	43.5 ± 6.3	43.7 ± 6.3
18:0	278.7 ± 26.1	267.3 ± 26.1	312.6 ± 26.1	277.4 ± 26.1	269.5 ± 26.1	284.4 ± 26.1	303.2 ± 26.1	268.2 ± 26.1
20:4n-6 ARA	124.4 ± 8.1 ^a^	94.8 ± 8.1 ^b^	90.1 ± 8.1 ^b^	105.7 ± 8.1 ^ab^	100.9 ± 8.1 ^b^	111.2 ± 8.1 ^ab^	105.9 ± 8.1 ^ab^	99.0 ± 8.1 ^b^
20:5n-3 EPA	33.7 ± 4.5	37.2 ± 4.5	31.2 ± 4.5	38.4 ± 4.5	27.0 ± 4.5	28.3 ± 4.5	38.1 ± 4.5	36.9 ± 4.5
20:3n-6	11.5 ± 0.5 ^a^	11.5 ± 0.5 ^a^	9.8 ± 0.5 ^b^	11.5 ± 0.5 ^a^	10.9 ± 0.5 ^ab^	11.7 ± 0.5 ^a^	10.9 ± 0.5 ^ab^	11.2 ± 0.5 ^ab^
20:4n-3	2.1 ± 0.3	2.0 ± 0.3	2.2 ± 0.3	1.9 ± 0.3	2.1 ± 0.3	1.5 ± 0.3	1.9 ± 0.3	2.1 ± 0.3
20:2n-6	2.0 ± 0.2 ^ab^	2.3 ± 0.2 ^a^	1.7 ± 0.2 ^b^	1.9 ± 0.2 ^ab^	2.0 ± 0.2 ^ab^	2.0 ± 0.2 ^ab^	1.8 ± 0.2 ^b^	1.9 ± 0.2 ^ab^
20:0	3.5 ± 0.3 ^ab^	3.4 ± 0.3 ^ab^	3.6 ± 0.3 ^ab^	3.1 ± 0.3 ^b^	3.3 ± 0.3 ^ab^	3.4 ± 0.3 ^ab^	4.0 ± 0.3 ^a^	3.2 ± 0.3 ^b^
22:5n-6 DPA-6	0.9 ± 0.2 ^ab^	1.1 ± 0.2 ^ab^	0.7 ± 0.2 ^b^	1.1 ± 0.2 ^ab^	1.4 ± 0.2 ^a^	1.1 ± 0.2 ^ab^	1.2 ± 0.2 ^ab^	0.9 ± 0.2 ^ab^
22:6n-3 DHA	18.7 ± 1.9 ^a^	13.1 ± 1.9 ^b^	12.7 ± 1.9 ^b^	16.7 ± 1.9 ^ab^	17.3 ± 1.9 ^ab^	16.0 ± 1.9 ^ab^	17.8 ± 1.9 ^ab^	17.9 ± 1.9 ^ab^
22:5n-3 DPA-3	37.5 ± 2.3 ^a^	36.2 ± 2.3 ^abc^	26.8 ± 2.3 ^d^	36.8 ± 2.3 ^ab^	29.8 ± 2.3 ^cd^	32.1 ± 2.3 ^abcd^	36.8 ± 2.3 ^ab^	30.3 ± 2.3 ^bcd^
22:0	5.7 ± 0.3	5.6 ± 0.3	5.4 ± 0.3	5.7 ± 0.3	5.5 ± 0.3	5.7 ± 0.3	6.0 ± 0.3	5.7 ± 0.3
23:0	7.4 ± 0.8 ^b^	8.2 ± 0.8 ^ab^	8.5 ± 0.8 ^ab^	9.8 ± 0.8 ^a^	9.2 ± 0.8 ^ab^	9.4 ± 0.8 ^ab^	8.5 ± 0.8 ^ab^	9.6 ± 0.8 ^ab^
24:0	5.4 ± 0.3	5.9 ± 0.3	5.6 ± 0.3	6.1 ± 0.3	6.0 ± 0.3	6.2 ± 0.3	5.8 ± 0.3	6.2 ± 0.3
Total FA	1743.8 ± 87.7	1768.3 ± 87.7	1719.2 ± 87.7	1777.5 ± 87.7	1655.1 ± 87.7	1721.1 ± 87.7	1823.7 ± 87.7	1682.4 ± 87.7
∑SFA	506.8 ± 42.9	511.9 ± 42.9	557.0 ± 42.9	522.7 ± 42.9	480.4 ± 42.9	521.8 ± 42.9	556.4 ± 42.9	510.2 ± 42.9
∑MUFA	387.9 ± 41.3	374.7 ± 41.3	405.1 ± 41.3	366.7 ± 41.3	387.0 ± 41.3	375.6 ± 41.3	435.7 ± 41.3	361.7 ± 41.3
∑PUFA	671.6 ± 22.4 ^a^	685.2 ± 22.4 ^a^	602.0 ± 22.4 ^b^	697.5 ± 22.4 ^a^	634.7 ± 22.4 ^ab^	656.1 ± 22.4 ^ab^	654.3 ± 22.4 ^ab^	667.2 ± 22.4 ^ab^
∑*n*-3 LC-PUFA	92.0 ± 7.7	88.4 ± 7.7	72.8 ± 7.7	93.8 ± 7.7	76.2 ± 7.7	77.9 ± 7.7	94.7 ± 7.7	87.2 ± 7.7
∑*n*-3 PUFA	125.2 ± 17.0	143.8 ± 17.0	127.2 ± 17.0	147.6 ± 17.0	110.1 ± 17.0	113.1 ± 17.0	149.2 ± 17.0	140.2 ± 17.0
∑*n*-6 PUFA	534.1 ± 29.3	530.8 ± 29.3	464.4 ± 29.3	541.1 ± 29.3	512.2 ± 29.3	532.7 ± 29.3	495.2 ± 29.3	518.4 ± 29.3
∑other FA	177.5 ± 18.4	196.5 ± 18.4	155.1 ± 18.4	190.4 ± 18.4	153.0 ± 18.4	167.5 ± 18.4	177.3 ± 18.4	143.3 ± 18.4
*n*-6/*n*-3	4.4 ± 0.7	3.9 ± 0.7	4.3 ± 0.7	3.8 ± 0.7	4.7 ± 0.7	4.8 ± 0.7	3.6 ± 0.7	4.3 ± 0.7

* Values within the same row bearing different superscripts differ (*p* < 0.05); all other abbreviations are as defined in Table 1, Table 2 and Table 3.

**Table 6:** Effect of pellet supplementation on total lipid percentage (g fat/100 g) and fatty acid contents (mg/100 g) in kidney of grazing prime lambs (LSM ± SE) *.

Items	Control		NOP		CO		RBO	
	CFP	Lucerne	CFP	Lucerne	CFP	Lucerne	CFP	Lucerne
Lipid percentage	2.8 ± 0.1	2.9 ± 0.1	2.8 ± 0.1	3.0 ± 0.1	2.9 ± 0.1	3.0 ± 0.1	2.9 ± 0.1	3.1 ± 0.1
14:0	6.4 ± 1.3	3.7 ± 1.3	5.9 ± 1.3	3.9 ± 1.3	5.0 ± 1.3	4.0 ± 1.3	7.1 ± 1.3	5.2 ± 1.3
15:0	4.5 ± 0.4 ^ab^	3.7 ± 0.4 ^b^	4.6 ± 0.4 ^ab^	4.1 ± 0.4 ^ab^	4.3 ± 0.4 ^ab^	4.4 ± 0.4 ^ab^	4.8 ± 0.4 ^a^	4.3 ± 0.4 ^ab^
16:1n-9c	4.9 ± 0.7	3.2 ± 0.7	4.4 ± 0.7	3.5 ± 0.7	4.2 ± 0.7	3.8 ± 0.7	4.4 ± 0.7	3.7 ± 0.7
16:1n-7c	6.4 ± 0.8	4.7 ± 0.8	6.2 ± 0.8	4.4 ± 0.8	4.8 ± 0.8	4.9 ± 0.8	6.3 ± 0.8	5.1 ± 0.8
16:0	318.9 ± 14.6 ^a^	272.2 ± 14.6 ^b^	304.4 ± 14.6 ^ab^	305.8 ± 14.6 ^ab^	286.8 ± 14.6 ^ab^	291.2 ± 14.6 ^ab^	305.2 ± 14.6 ^ab^	311.7 ± 14.6 ^ab^
17:0	18.4 ± 0.8 ^abc^	18.1 ± 0.8 ^abc^	18.0 ± 0.8 ^bc^	20.4 ± 0.8 ^a^	17.3 ± 0.8 ^c^	19.5 ± 0.8 ^abc^	18.9 ± 0.8 ^abc^	19.7 ± 0.8 ^ab^
18:2n-6 LA	228.0 ± 14.2	215.3 ± 14.2	221.0 ± 14.2	250.0 ± 14.2	240.5 ± 14.2	250.3 ± 14.2	229.2 ± 14.2	228.1 ± 14.2
18:3n-3 ALA	23.8 ± 4.7	29.4 ± 4.7	33.0 ± 4.7	28.0 ± 4.7	22.2 ± 4.7	23.0 ± 4.7	34.7 ± 4.7	31.7 ± 4.7
18:1n-9c	240.8 ± 15.5	200.1 ± 15.5	226.3 ± 15.5	213.8 ± 15.5	226.3 ± 15.5	219.5 ± 15.5	232.9 ± 15.5	211.9 ± 15.5
18:1n-7c	34.0 ± 2.6	27.8 ± 2.6	27.5 ± 2.6	30.1 ± 2.6	30.1 ± 2.6	30.5 ± 2.6	27.5 ± 2.6	27.2 ± 2.6
18:1n-7t	37.1 ± 6.6	34.3 ± 6.6	31.6 ± 6.6	36.5 ± 6.6	39.6 ± 6.6	37.9 ± 6.6	39.2 ± 6.6	36.6 ± 6.6
18:0	319.0 ± 16.0	291.3 ± 16.0	308.4 ± 16.0	336.3 ± 16.0	310.7 ± 16.0	301.9 ± 16.0	324.5 ± 16.0	318.6 ± 16.0
20:4n-6 ARA	236.9 ± 16.9 ^a^	184.8 ± 16.9 ^b^	202.9 ± 16.9 ^ab^	221.7 ± 16.9 ^ab^	214.6 ± 16.9 ^ab^	218.0 ± 16.9 ^ab^	210.7 ± 16.9 ^ab^	218.6 ± 16.9 ^ab^
20:5n-3 EPA	57.2 ± 11.7	70.2 ± 11.7	70.1 ± 11.7	76.2 ± 11.7	49.9 ± 11.7	49.8 ± 11.7	75.5 ± 11.7	68.9 ± 11.7
20:3n-6	15.5 ± 1.4 ^b^	16.2 ± 1.4 ^b^	15.2 ± 1.4^b^	20.4 ± 1.4 ^a^	15.1 ± 1.4 ^b^	16.2 ± 1.4 ^b^	15.5 ± 1.4 ^b^	18.3 ± 1.4 ^ab^
20:4n-3	2.7 ± 0.6	3.2 ± 0.6	4.2 ± 0.6	2.8 ± 0.6	2.5 ± 0.6	3.0 ± 0.6	3.8 ± 0.6	4.3 ± 0.6
20:2n-6	4.1 ± 0.6	4.6 ± 0.6	4.9 ± 0.6	5.4 ± 0.6	4.6 ± 0.6	5.8 ± 0.6	4.2 ± 0.6	5.8 ± 0.6
20:0	5.4 ± 0.4	5.0 ± 0.4	5.5 ± 0.4	5.5 ± 0.4	4.9 ± 0.4	5.8 ± 0.4	5.4 ± 0.4	5.6 ± 0.4
22:5n-6 DPA-6	1.5 ± 0.1 ^a^	0.9 ± 0.1 ^b^	1.1 ± 0.1 ^ab^	1.0 ± 0.1 ^b^	1.5 ± 0.1 ^a^	1.0 ± 0.1 ^b^	1.1 ± 0.1 ^ab^	1.0 ± 0.1 ^b^
22:6n-3 DHA	51.4 ± 5.4 ^ab^	37.7 ± 5.4 ^b^	47.3 ± 5.4 ^ab^	49.9 ± 5.4 ^ab^	55.6 ± 5.4 ^a^	45.8 ± 5.4 ^ab^	53.0 ± 5.4 ^ab^	53.7 ± 5.4 ^ab^
22:5n-3 DPA-3	66.1 ± 5.8 ^b^	72.4 ± 5.8 ^ab^	71.6 ± 5.8 ^ab^	84.4 ± 5.8 ^a^	63.7 ± 5.8 ^b^	64.5 ± 5.8 ^b^	68.5 ± 5.8 ^ab^	72.1 ± 5.8 ^ab^
22:0	29.5 ± 1.9 ^ab^	26.2 ± 1.9 ^b^	30.1 ± 1.9 ^ab^	30.7 ± 1.9 ^ab^	28.2 ± 1.9 ^ab^	31.8 ± 1.9 ^a^	29.2 ± 1.9 ^ab^	29.8 ± 1.9 ^ab^
23:0	9.0 ± 0.7 ^ab^	8.5 ± 0.7 ^b^	8.9 ± 0.7 ^ab^	10.3 ± 0.7 ^ab^	9.3 ± 0.7 ^ab^	10.4 ± 0.7 ^a^	9.6 ± 0.7 ^ab^	10.0 ± 0.7 ^ab^
24:0	31.5 ± 2.6	30.1 ± 2.6	32.4 ± 2.6	33.8 ± 2.6	32.8 ± 2.6	32.1 ± 2.6	30.9 ± 2.6	33.0 ± 2.6
Total FA	1924.5 ± 75.8 ^ab^	1710.2 ± 75.8 ^b^	1852.6 ± 75.8 ^ab^	1953.3 ± 75.8 ^a^	1840.2 ± 75.8 ^ab^	1850.3 ± 75.8 ^ab^	1905.0 ± 75.8 ^ab^	1904.2 ± 75.8 ^ab^
∑SFA	742.6 ± 32.2	658.6 ± 32.2	718.1 ± 32.2	750.9 ± 32.2	699.3 ± 32.2	701.2 ± 32.2	735.6 ± 32.2	737.8 ± 32.2
∑MUFA	413.2 ± 25.2	347.6 ± 25.2	379.0 ± 25.2	383.5 ± 25.2	392.6 ± 25.2	385.1 ± 25.2	393.0 ± 25.2	372.4 ± 25.2
∑PUFA	706.0 ± 26.3 ^ab^	650.1 ± 26.3 ^b^	688.5 ± 26.3 ^ab^	755.2 ± 26.3 ^a^	685.8 ± 26.3 ^ab^	696.6 ± 26.3 ^ab^	712.2 ± 26.3 ^ab^	722.4 ± 26.3 ^ab^
∑*n*-3 LC-PUFA	177.3 ± 19.6	183.6 ± 19.6	193.1 ± 19.6	213.2 ± 19.6	171.6 ± 19.6	163.2 ± 19.6	200.7 ± 19.6	202.2 ± 19.6
∑*n*-3 PUFA	201.1 ± 23.2	213.0 ± 23.2	226.2 ± 23.2	241.3 ± 23.2	193.8 ± 23.2	186.2 ± 23.2	235.5 ± 23.2	234.0 ± 23.2
∑*n*-6 PUFA	493.2 ± 26.7 ^ab^	427.2 ± 26.7 ^b^	451.9 ± 26.7 ^ab^	505.4 ± 26.7 ^a^	482.6 ± 26.7 ^ab^	500.4 ± 26.7 ^ab^	466.4 ± 26.7 ^ab^	479.0 ± 26.7 ^ab^
∑other FA	62.7 ± 4.3 ^ab^	53.9 ± 4.3 ^b^	67.0 ± 4.3 ^a^	63.7 ± 4.3 ^ab^	62.5 ± 4.3 ^ab^	67.5 ± 4.3 ^a^	64.2 ± 4.3 ^ab^	71.6 ± 4.3 ^a^
*n*-6/*n*-3	2.5 ± 0.3	2.1 ± 0.3	2.2 ± 0.3	2.2 ± 0.3	2.5 ± 0.3	2.7 ± 0.3	2.0 ± 0.3	2.2 ± 0.3

* Values within the same row bearing different superscripts differ (*p* < 0.05); all other abbreviations are as defined in Table 1, Table 2 and Table 3.

**Table 7:** Effect of different pasture types on total lipid percentage (g fat/100 g) and fatty acid contents (mg/100 g) in liver, kidney, heart and *Longissimus dorsi* muscle of prime lambs (LSM ± SE) *.

Items	Liver		*p*	Kidney		*p*	Heart		*p*	Muscle		*p*
	CFP	Lucerne		CFP	Lucerne		CFP	Lucerne		CFP	Lucerne	
Lipid percentage	6.6 ± 0.2	6.8 ± 0.2	0.460	2.9 ± 0.1	3.0 ± 0.1	0.055	2.4 ± 0.1	2.3 ± 0.1	0.865	2.9 ± 0.3	3.1 ± 0.3	0.697
14:0	28.3 ± 2.5	22.0 ± 2.5	0.089	6.1 ± 0.7 ^a^	4.2 ± 0.7 ^b^	0.047	6.8 ± 1.1	6.0 ± 1.1	0.608	48.8 ± 6.9	52.3 ± 6.9	0.727
15:0	15.1 ± 0.6	13.2 ± 0.6	0.029	4.6 ± 0.2	4.1 ± 0.2	0.097	2.9 ± 0.2	2.9 ± 0.2	0.971	6.5 ± 0.8	7.5 ± 0.8	0.381
16:1n-9c	24.8 ± 2.0	21.4 ± 2.0	0.245	4.5 ± 0.4	3.5 ± 0.4	0.088	2.9 ± 0.2	2.6 ± 0.2	0.374	6.0 ± 0.7	6.8 ± 0.7	0.361
16:1n-7c	39.6 ± 5.2	30.2 ± 5.2	0.209	5.9 ± 0.4 ^a^	4.7 ± 0.4 ^b^	0.049	5.6 ± 0.4	4.9 ± 0.4	0.260	29.6 ± 3.8	34.9 ± 3.8	0.347
16:0	762.5 ± 41.7	722.8 ± 41.7	0.508	303.8 ± 7.3	295.2 ± 7.3	0.413	187.2 ± 6.7	194.2 ± 6.7	0.470	527.2 ± 56.9	637.6 ± 56.9	0.183
17:0	53.1 ± 1.9	58.3 ± 1.9	0.063	18.1 ± 0.4 ^b^	19.4 ± 0.4 ^a^	0.031	14.0 ± 0.8	15.1 ± 0.8	0.341	22.4 ± 2.8	29.9 ± 2.8	0.064
18:2n-6 LA	377.7 ± 24.2	398.9 ± 24.2	0.542	229.7 ± 7.1	235.9 ± 7.1	0.540	377.8 ± 12.2	408.5 ± 12.2	0.088	118.3 ± 4.4 ^b^	145.3 ± 4.4 ^a^	0.000
18:3n-3 ALA	139.6 ± 11.4	155.8 ± 11.4	0.324	28.4 ± 2.3	28.0 ± 2.3	0.912	43.7 ± 5.1	49.1 ± 5.1	0.460	43.1 ± 2.8 ^b^	54.1 ± 2.8 ^a^	0.009
18:1n-9c	985.9 ± 74.2	852.3 ± 74.2	0.215	231.6 ± 7.7	211.3 ± 7.7	0.076	226.5 ± 17.3	195.0 ± 17.3	0.211	905.4 ± 98.5	1108.3 ± 98.5	0.158
18:1n-7c	77.0 ± 7.5	69.7 ± 7.5	0.495	29.8 ± 1.3	28.9 ± 1.3	0.632	36.8 ± 1.7	36.4 ± 1.7	0.864	39.3 ± 3.8	48.3 ± 3.8	0.103
18:1n-7t	237.9 ± 21.7	227.2 ± 21.7	0.732	36.9 ± 3.3	36.3 ± 3.3	0.906	45.4 ± 3.1	46.5 ± 3.1	0.800	66.1 ± 9.2	91.2 ± 9.2	0.064
18:0	876.0 ± 32.9	890.1 ± 32.9	0.764	315.6 ± 8.0	312.0 ± 8.0	0.752	291.0 ± 13.1	274.3 ± 13.1	0.376	397.6 ± 37.7	453.9 ± 37.7	0.302
20:4n-6 ARA	228.2 ± 11.6	223.9 ± 11.6	0.794	216.2 ± 8.4	210.7 ± 8.4	0.650	105.3 ± 4.0	102.7 ± 4.0	0.646	35.3 ± 1.5	36.4 ± 1.5	0.582
20:5n-3 EPA	90.8 ± 7.2	106.5 ± 7.2	0.139	63.2 ± 5.8	66.3 ± 5.8	0.709	32.5 ± 2.2	35.2 ± 2.2	0.409	21.7 ± 1.0	23.8 ± 1.0	0.140
20:3n-6	32.7 ± 1.7	38.7 ± 1.7	0.019	15.3 ± 0.7 ^b^	17.7 ± 0.7 ^a^	0.024	10.7 ± 0.3	11.5 ± 0.3	0.055	6.2 ± 0.2 ^b^	7.4 ± 0.2 ^a^	0.001
20:4n-3	10.0 ± 0.7	11.5 ± 0.7	0.112	3.3 ± 0.3	3.3 ± 0.3	0.968	2.1 ± 0.2	1.9 ± 0.2	0.400	1.9 ± 0.1	2.0 ± 0.1	0.802
20:2n-6	5.9 ± 0.4	6.3 ± 0.4	0.513	4.4 ± 0.3 ^b^	5.4 ± 0.3 ^a^	0.043	1.9 ± 0.1	2.1 ± 0.1	0.170	1.5 ± 0.1	1.8 ± 0.1	0.073
20:0	6.1 ± 0.4	5.3 ± 0.4	0.143	5.3 ± 0.2	5.5 ± 0.2	0.500	3.6 ± 0.1	3.3 ± 0.1	0.114	3.6 ± 0.3	3.7 ± 0.3	0.713
22:5n-6 DPA-6	8.3 ± 1.1	5.5 ± 1.1	0.081	1.3 ± 0.1 ^a^	1.0 ± 0.1 ^b^	0.003	1.0 ± 0.1	1.0 ± 0.1	0.922	1.3 ± 0.1	1.2 ± 0.1	0.454
22:6n-3 DHA	169.7 ± 13.1	165.8 ± 13.1	0.833	51.8 ± 2.7	47.6 ± 2.7	0.279	16.6 ± 1.0	15.9 ± 1.0	0.607	6.2 ± 0.4	6.7 ± 0.4	0.363
22:5n-3 DPA-3	208.2 ± 10.1	235.9 ± 10.1	0.064	67.4 ± 2.9	73.3 ± 2.9	0.166	32.7 ± 1.2	33.8 ± 1.2	0.496	20.6 ± 0.5 ^b^	22.6 ± 0.5 ^a^	0.015
22:0	8.6 ± 0.2	7.9 ± 0.2	0.057	29.2 ± 0.9	29.6 ± 0.9	0.775	5.6 ± 0.1	5.7 ± 0.1	0.863	1.4 ± 0.1	1.5 ± 0.1	0.208
23:0	18.8 ± 1.1	21.0 ± 1.1	0.181	9.2 ± 0.3	9.8 ± 0.3	0.203	8.4 ± 0.4	9.2 ± 0.4	0.138	1.6 ± 0.1 ^b^	1.8 ± 0.1 ^a^	0.022
24:0	16.1 ± 0.5	15.4 ± 0.5	0.288	31.9 ± 1.3	32.2 ± 1.3	0.867	5.7 ± 0.1	6.1 ± 0.1	0.067	2.0 ± 0.1	2.1 ± 0.1	0.089
Total FA	4829.7 ± 223.0	4680.3 ± 223.0	0.640	1880.6 ± 37.9	1854.5 ± 37.9	0.631	1735.4 ± 43.8	1737.3 ± 43.8	0.976	2505.3 ± 233.9	2987.9 ± 233.9	0.158
∑SFA	1784.5 ± 71.5	1756.0 ± 71.5	0.781	723.9 ± 16.1	712.1 ± 16.1	0.610	525.1 ± 21.5	516.6 ± 21.5	0.782	1010.9 ± 104.0	1190.4 ± 104.0	0.234
∑MUFA	1582.7 ± 115.5	1405.7 ± 115.5	0.290	394.4 ± 12.6	372.2 ± 12.6	0.223	403.9 ± 20.7	369.7 ± 20.7	0.254	1140.3 ± 121.4	1401.9 ± 121.4	0.141
∑PUFA	1333.6 ± 48.3	1404.9 ± 48.3	0.306	698.1 ± 13.2	706.1 ± 13.2	0.673	640.6 ± 11.2 ^b^	676.5 ± 11.2 ^a^	0.033	266.9 ± 8.6 ^b^	312.4 ± 8.6 ^a^	0.001
∑*n*-3 LC-PUFA	478.8 ± 24.8	519.7 ± 24.8	0.255	185.7 ± 9.8	190.5 ± 9.8	0.729	83.9 ± 3.9	86.8 ± 3.9	0.603	50.4 ± 1.8	55.1 ± 1.8	0.074
∑*n*-3 PUFA	626.4 ± 34.1	681.3 ± 34.1	0.266	214.1 ± 11.6	218.6 ± 11.6	0.788	127.9 ± 8.5	136.2 ± 8.5	0.500	94.0 ± 3.8 ^b^	110.1 ± 3.8 ^a^	0.007
∑*n*-6 PUFA	683.3 ± 38.0	702.6 ± 38.0	0.722	473.5 ± 13.4	478.0 ± 13.4	0.816	501.5 ± 14.6	530.8 ± 14.6	0.170	166.1 ± 5.4 ^b^	196.2 ± 5.4 ^a^	0.001
∑other FA	127.6 ± 8.1	113.1 ± 8.1	0.220	64.1 ± 2.1	64.2 ± 2.1	0.979	165.7 ± 9.2	174.4 ± 9.2	0.510	86.1 ± 6.4	82.2 ± 6.4	0.674
*n*-6/*n*-3	1.1 ± 0.1	1.1 ± 0.1	0.957	2.3 ± 0.1	2.3 ± 0.1	0.928	4.2 ± 0.3	4.2 ± 0.3	0.929	1.8 ± 0.0	1.9 ± 0.0	0.788

* Values within the same row bearing different superscripts differ (*p* < 0.05); all other abbreviations are as defined in Table 1, Table 2 and Table 3.

## References

[1] Calder P.C. (2009). Polyunsaturated fatty acids and inflammatory processes: New twists in an old tale. Biochimie.

[2] Cao Y., Lu L., Liang J., Liu M., Li X.C., Sun R.R., Zheng Y., Zhang P.Y. (2015). Omega-3 fatty acids and primary and secondary prevention of cardiovascular disease. Cell Biochem. Biophys..

[3] Leslie M.A., Cohen D.J.A., Liddle D.M., Robinson L.E., Ma D.W.L. (2015). A review of the effect of omega-3 polyunsaturated fatty acids on blood triacylglycerol levels in normolipidemic and borderline hyperlipidemic individuals. Lipids Health Dis..

[4] Cabo-Garcia L., Achon-Tunon M., Gonzalez-Gonzalez M.P. (2015). The influence of polyunsaturated fatty acids in the prevention and promotion of cancer. Nutr. Hosp..

[5] Fu Y.Q., Zheng J.S., Yang B., Li D. (2015). Effect of individual omega-3 fatty acids on the risk of prostate cancer: A systematic review and dose-response meta-analysis of prospective cohort studies. J. Epidemiol..

[6] Manzi L., Costantini L., Molinari R., Merendino N. (2015). Effect of dietary omega-3 polyunsaturated fatty acid DHA on glycolytic enzymes and Warburg phenotypes in cancer. Biomed. Res. Int..

[7] Nabavi S.F., Bilotto S., Russo G.L., Orhan I.E., Habtemariam S., Daglia M., Devi K.P., Loizzo M.R., Tundis R., Nabavi S.M. (2015). Omega-3 polyunsaturated fatty acids and cancer: Lessons learned from clinical trials. Cancer Metast. Rev..

[8] Sheppard K.W., Cheatham C.L. (2018). Omega-6/omega-3 fatty acid intake of children and older adults in the U.S.: Dietary intake in comparison to current dietary recommendations and the Healthy Eating Index. Lipids Health Dis..

[9] Pittaway J., Chuang L., Ahuja K., Beckett J., Glew R., Ball M. (2015). Omega-3 dietary fatty acid status of healthy older adults in Tasmania, Australia: An observational study. J. Nutr. Health Aging.

[10] Nichols P.D., Petrie J., Singh S. (2010). Long-chain omega-3 oils–an update on sustainable sources. Nutrients.

[11] Howe P., Buckley J., Meyer B. (2007). Long-chain omega-3 fatty acids in red meat. Nutr. Diet..

[12] ABARES Agricultural Commodities and Trade Data. http://www.agriculture.gov.au/abares/research-topics/agricultural-commodities/agricultural-commodities-trade-data#2017.

[13] Wong L., Selvanathan E.A., Selvanathan S. (2015). Modelling the meat consumption patterns in Australia. Econ. Model..

[14] De Brito G.F., Ponnampalam E.N., Hopkins D.L. (2017). The effect of extensive feeding systems on growth rate, carcass traits, and meat quality of finishing lambs. Compr. Rev. Food Sci. F..

[15] Lolicato S., Rumball W. (1994). Past and present improvement of cocksfoot (*Dactylis glomerata* L.) in Australia and New Zealand. N. Z. J. Agric. Res..

[16] Clark S.G., Nie Z.N., Culvenor R.A., Harris C.A., Hayes R.C., Li G.D., Norton M.R., Partington D.L. (2016). Field evaluation of cocksfoot, tall fescue and phalaris for dry marginal environments of south-eastern australia. 1. Establishment and herbage production. J. Agron. Crop Sci..

[17] Clapham W.M., Foster J.G., Neel J.P., Fedders J.M. (2005). Fatty acid composition of traditional and novel forages. J. Agric. Food Chem..

[18] Casey N.H., van Niekerk W.A., Spreeth E.B. (1988). Fatty acid composition of subcutaneous fat of sheep grazed on eight different pastures. Meat Sci..

[19] Chikwanha O.C., Vahmani P., Muchenje V., Dugan M.E.R., Mapiye C. (2018). Nutritional enhancement of sheep meat fatty acid profile for human health and wellbeing. Food Res. Int..

[20] Ponnampalam E.N., Butler K.L., Jacob R.H., Pethick D.W., Ball A.J., Hocking Edwards J.E., Geesink G., Hopkins D.L. (2014). Health beneficial long chain omega-3 fatty acid levels in Australian lamb managed under extensive finishing systems. Meat Sci..

[21] Humphries A.W. (2012). Future applications of lucerne for efficient livestock production in southern Australia. Crop Pasture Sci..

[22] Ponnampalam E.N., Linden N.P., Mitchell M.L., Hopkins D.L., Jacobs J.L. (2017). Production systems to deliver premium grade lambs to the growing international and Australian markets. Small Ruminant Res..

[23] Fraser M.D., Speijers M.H.M., Theobald V.J., Fychan R., Jones R. (2004). Production performance and meat quality of grazing lambs finished on red clover, lucerne or perennial ryegrass swards. Grass Forage Sci..

[24] Boughalmi A., Araba A. (2016). Effect of feeding management from grass to concentrate feed on growth, carcass characteristics, meat quality and fatty acid profile of Timahdite lamb breed. Small Ruminant Res..

[25] Turner K.E., Belesky D.P., Cassida K.A., Zerby H.N. (2014). Carcass merit and meat quality in Suffolk lambs, Katahdin lambs, and meat-goat kids finished on a grass–legume pasture with and without supplementation. Meat Sci..

[26] Nguyen D.V., Flakemore A.R., Otto J.R., Ives S.W., Smith R.W., Nichols P.D., Malau-Aduli A.E.O. (2017). Nutritional value and sensory characteristics of meat eating quality of Australian prime lambs supplemented with pelleted canola and flaxseed oils: Fatty acid profiles of muscle and adipose tissues. Int. Med. Rev..

[27] Goffman F.D., Pinson S., Bergman C. (2003). Genetic diversity for lipid content and fatty acid profile in rice bran. J. Am. Oil Chem. Soc..

[28] Lunsin R., Wanapat M., Yuangklang C., Rowlinson P. (2012). Effect of rice bran oil supplementation on rumen fermentation, milk yield and milk composition in lactating dairy cows. Livest. Sci..

[29] Bhatt R.S., Sahoo A., Karim S.A., Agrawal A.R. (2016). Effects of calcium soap of rice bran oil fatty acids supplementation alone and with DL-α-tocopherol acetate in lamb diets on performance, digestibility, ruminal parameters and meat quality. J. Anim. Physiol. Anim. Nutr..

[30] Umaraw P., Pathak V., Rajkumar V., Verma A.K., Singh V.P., Verma A.K. (2015). Assessment of fatty acid and mineral profile of Barbari kid in longissimus lumborum muscle and edible byproducts. Small Ruminant Res..

[31] Bester M., Schönfeldt H.C., Pretorius B., Hall N. (2018). The nutrient content of selected South African lamb and mutton organ meats (offal). Food Chem..

[32] Nguyen D.V., Le V.H., Nguyen Q.V., Malau-Aduli B.S., Nichols P.D., Malau-Aduli A.E.O. (2017). Omega–3 long-chain fatty acids in the heart, kidney, liver and plasma metabolite profiles of australian prime lambs supplemented with pelleted canola and flaxseed oils. Nutrients.

[33] Malau-Aduli A.E.O., Holman B.W.B., Kashani A., Nichols P.D. (2016). Sire breed and sex effects on the fatty acid composition and content of heart, kidney, liver, adipose and muscle tissues of purebred and first-cross prime lambs. Anim. Prod. Sci..

[34] Amaral D.S., Silva F.A.P., Bezerra T.K.A., Arcanjo N.M.O., Guerra I.C.D., Dalmás P.S., Madruga M.S. (2015). Effect of storage time and packaging on the quality of lamb pâté prepared with ‘variety meat’. Food Packaging Shelf.

[35] Bligh E.G., Dyer W.J. (1959). A rapid method of total lipid extraction and purification. Can. J. Biochem. Physiol..

[36] Miller M.R., Nichols P.D., Barnes J., Davies N.W., Peacock E.J., Carter C.G. (2006). Regiospecificity profiles of storage and membrane lipids from the gill and muscle tissue of Atlantic salmon (*Salmo salar* L.) grown at elevated temperature. Lipids.

[37] Flakemore A.R., Malau-Aduli B.S., Nichols P.D., Malau-Aduli A.E.O. (2017). Omega-3 fatty acids, nutrient retention values, and sensory meat eating quality in cooked and raw Australian lamb. Meat Sci..

[38] Clayton E.H., Nugent T., Nicholls C. (2014). Graham Centre Monograph No. 4: Long-Chain Omega-3 Polyunsaturated Fatty Acids in Ruminant Nutrition: Benefits to Animals and Humans.

[39] (2009). SAS. Statistical Analysis System. SAS/STAT User’s Guide: Statistics.

[40] Meľuchová B., Blaško J., Kubinec R., Górová R., Dubravská J., Margetín M., Soják L. (2008). Seasonal variations in fatty acid composition of pasture forage plants and CLA content in ewe milk fat. Small Ruminant Res..

[41] Garcia P.T., Pordomingo A., Perez C.D., Rios M.D., Sancho A.M., Volpi Lagreca G., Casal J.J. (2016). Influence of cultivar and cutting date on the fatty acid composition of forage crops for grazing beef production in Argentina. Grass Forage Sci..

[42] Wiking L., Theil P.K., Nielsen J.H., Sorensen M.T. (2010). Effect of grazing fresh legumes or feeding silage on fatty acids and enzymes involved in the synthesis of milk fat in dairy cows. J. Dairy Res..

[43] Glasser F., Doreau M., Maxin G., Baumont R. (2013). Fat and fatty acid content and composition of forages: A meta-analysis. Anim. Feed Sci. Tech..

[44] Nguyen D.V., Malau-Aduli B.S., Cavalieri J., Nichols P.D., Malau-Aduli A.E.O. (2018). Supplementation with plant-derived oils rich in omega-3 polyunsaturated fatty acids for lamb production. Veterinary Anim. Sci..

[45] Ponnampalam E.N., Burnett V.F., Norng S., Warner R.D., Jacobs J.L. (2012). Vitamin E and fatty acid content of lamb meat from perennial pasture or annual pasture systems with supplements. Anim. Prod. Sci..

[46] Fruet A.P.B., Trombetta F., Stefanello F.S., Speroni C.S., Donadel J.Z., De Souza A.N.M., Rosado Júnior A., Tonetto C.J., Wagner R., De Mello A. (2018). Effects of feeding legume-grass pasture and different concentrate levels on fatty acid profile, volatile compounds, and off-flavor of the *M. longissimus thoracis*. Meat Sci..

[47] Raes K., De Smet S., Demeyer D. (2004). Effect of dietary fatty acids on incorporation of long chain polyunsaturated fatty acids and conjugated linoleic acid in lamb, beef and pork meat: A review. Anim. Feed Sci. Tech..

[48] Ponnampalam E.N., Butler K.L., Pearce K.M., Mortimer S.I., Pethick D.W., Ball A.J., Hopkins D.L. (2014). Sources of variation of health claimable long chain omega-3 fatty Australian lamb slaughtered at similar weights. Meat Sci..

[49] Kashani A., Holman B.W.B., Nichols P.D., Malau-Aduli A.E.O. (2015). Effect of level of Spirulina supplementation on the fatty acid compositions of adipose, muscle, heart, kidney and liver tissues in Australian dual-purpose lambs. Ann. Anim. Sci..

[50] Simopoulos A.P. (2008). The importance of the omega-6/omega-3 fatty acid ratio in cardiovascular disease and other chronic diseases. Exp. Biol. Med..

[51] Nutrition Information User Guide to Standard 1.2.8—Nutrition Information Requirements Part B—Nutrition Claims. http://www.foodstandards.gov.au/code/userguide/Documents/Userguide_Nutrition%20Claims_PartB_March12.pdf.

[52] Johansen M., Lund P., Weisbjerg M.R. (2017). Feed intake and milk production in dairy cows fed different grass and legume species: A meta-analysis. Animal.

[53] Kitessa S., Liu S., Briegel J., Pethick D., Gardner G., Ferguson M., Allingham P., Nattrass G., McDonagh M., Ponnampalam E. (2010). Effects of intensive or pasture finishing in spring and linseed supplementation in autumn on the omega-3 content of lamb meat and its carcass distribution. Anim. Prod. Sci..

[54] Bessa R.J.B., Alves S.P., Santos-Silva J. (2015). Constraints and potentials for the nutritional modulation of the fatty acid composition of ruminant meat. Eur. J. Lipid Sci. Tech..

